# Machine Learning in 3D and 4D Printing of Polymer Composites: A Review

**DOI:** 10.3390/polym16223125

**Published:** 2024-11-08

**Authors:** Ivan Malashin, Igor Masich, Vadim Tynchenko, Andrei Gantimurov, Vladimir Nelyub, Aleksei Borodulin, Dmitry Martysyuk, Andrey Galinovsky

**Affiliations:** 1 Artificial Intelligence Technology Scientific and Education Center, Bauman Moscow State Technical University, 105005 Moscow, Russia; imasich@emtc.ru (I.M.); agantimurov@emtc.ru (A.G.);; 2 Scientific Department, Far Eastern Federal University, 690922 Vladivostok, Russia; 3 Center NTI “Digital Materials Science: New Materials and Substances”, Bauman Moscow State Technical University, 105005 Moscow, Russia; dmart9945@mail.ru (D.M.); a_galinovskiy@bmstu.ru (A.G.)

**Keywords:** machine learning, polymer composites, 3D printing, 4D printing, design optimization

## Abstract

The emergence of 3D and 4D printing has transformed the field of polymer composites, facilitating the fabrication of complex structures. As these manufacturing techniques continue to progress, the integration of machine learning (ML) is widely utilized to enhance aspects of these processes. This includes optimizing material properties, refining process parameters, predicting performance outcomes, and enabling real-time monitoring. This paper aims to provide an overview of the recent applications of ML in the 3D and 4D printing of polymer composites. By highlighting the intersection of these technologies, this paper seeks to identify existing trends and challenges, and outline future directions.

## 1. Introduction

In recent years, 3D and 4D printing of polymer composites have gained attention across various industries, ranging from healthcare [1,2] to aerospace [3,4]. Simultaneously, machine learning (ML) is emerging as a key tool for optimizing additive manufacturing (AM) processes, offering methods to predict material properties, improve printing parameters, and enhance overall efficiency. Despite the growing interest in this intersection, the number of studies combining ML with 4D printing (4DP) of polymer composites remains relatively limited, with most publications focusing on specific applications of ML. According to data from Scopus, there are currently 47 publications that include the terms “machine learning”, “polymer composites”, and “3D or 4D printing”. Notably, the year 2024 has seen the highest output, with 21 publications, suggesting a significant increase in research activity in this area.

**Figure 1:**
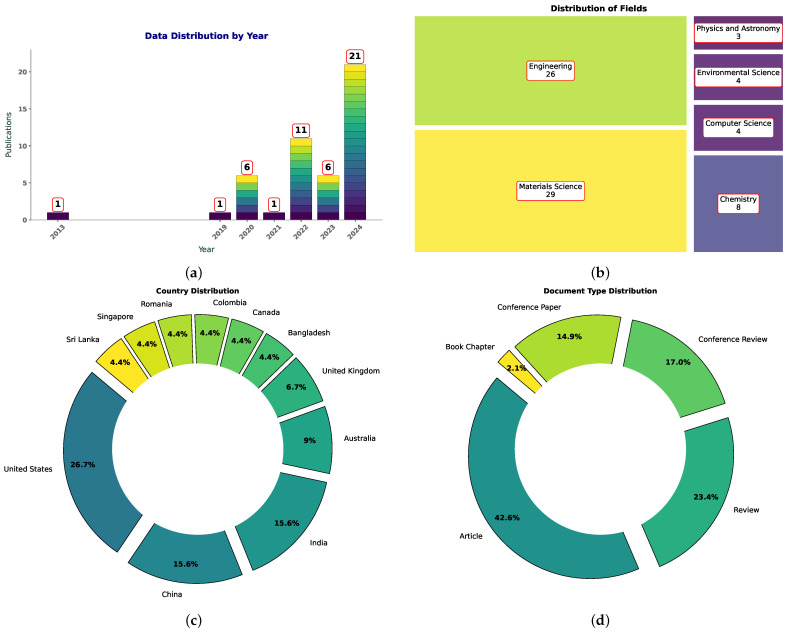
Data visualizations illustrating various distributions: (**a**) annual publication counts over the years, (**b**) distribution of fields in the analyzed dataset, (**c**) geographical distribution of contributions by country, and (**d**) distribution of document types. According to Scopus data.

Figure 1 presents visualizations of the analysis of publications related to machine learning, polymer composites, and 3D/4D printing based on Scopus data. Figure 1a illustrates the changes in the number of publications on this topic over time, providing insights into the relevance and growing interest in this research area. Figure 1b shows the distribution of these publications across various fields, highlighting the most active research directions. Figure 1c depicts the geographical distribution of publications from different countries, revealing the regions that are actively contributing to this field of study. Finally, Figure 1d presents the types of documents prevalent in this area, whether they are articles, conference papers, or other formats.

The aim of this review is to systematically analyze the existing literature, discuss current advancements, and identify key challenges in the application of ML to 3DP and 4DP of polymer composites. Additionally, this review provides recommendations for future research to integrate these technologies. The structure of the paper covers the role of ML in material enhancement, process optimization, property prediction, and quality monitoring, as well as a discussion of the future prospects in this field.

To facilitate the reader’s understanding of these topics, the paper presents a structured outline. It begins with an overview of 3D and 4D printing technologies in Section 2, laying the groundwork for understanding various additive manufacturing methods. This includes examining material extrusion (Section 2.1) and stereolithography (Section 2.2), followed by an exploration of selective laser sintering (Section 2.3) and digital light processing (Section 2.4). Next, the paper delves into jetting technologies (Section 2.5) and direct ink writing (Section 2.6), highlighting their unique features and applications. The concept of 4D printing is introduced (Section 2.7), showcasing its potential for creating adaptive materials. The properties of polymer composites used in these technologies are analyzed (Section 3), followed by a discussion on the applications of machine learning in both 3D (Section 4) and 4D printing (Section 5) of polymer composites. This leads into a section on transforming polymer composites into ceramics and other materials (Section 6), addressing relevant processes and implications. The paper also discusses challenges and limitations associated with these technologies (Section 7), providing a critical perspective on their current state. It concludes with future directions for research and development (Section 8), emphasizing the importance of continued innovation in additive manufacturing, before wrapping up with a summary of the key findings (Section 9).

## 2. 3D and 4D Printing: An Overview

Three-dimensional printing (3DP) is a technology that enables the creation of complex structures by layering materials according to digital models. The most common techniques used in 3DP of polymer composites include fused deposition modeling (FDM), stereolithography (SLA), and selective laser sintering (SLS). These methods allow for high precision and customization in the production of functional parts [5], which makes 3DP suitable for a wide range of applications, from rapid prototyping to the production of end-use products in industries like aerospace, automotive, healthcare, and consumer goods.

Table 1 outlines key 3DP and extrusion techniques, including their processes, common materials, potential applications, advantages, and associated challenges.

Four-dimensional printing extends the capabilities of 3DP by introducing time as a fourth dimension (all techniques are summarized in Table 2). It involves the fabrication of smart materials or structures that can change shape, properties, or behavior over time in response to external stimuli, such as heat, moisture, or light [30].

**Figure 2:**
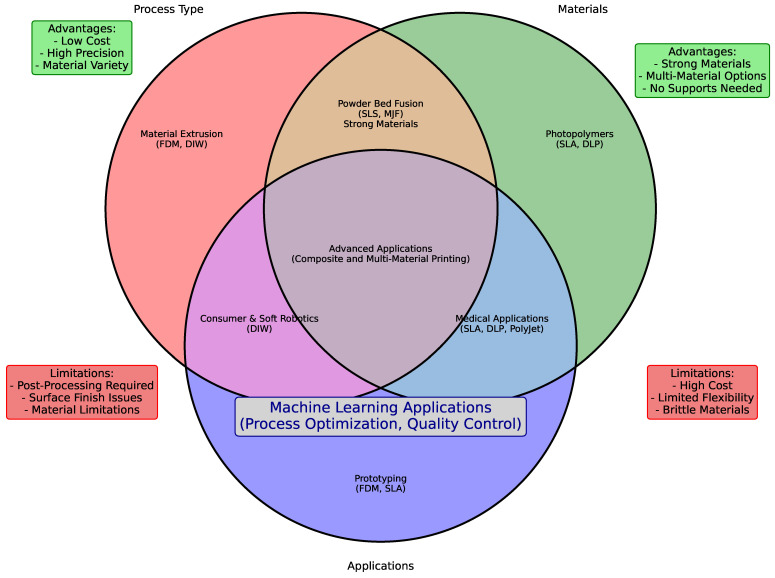
Venn diagram illustrating key aspects of various 3DP techniques.

These dynamic transformations open up possibilities for creating self-assembling systems, adaptive materials, and responsive structures, which are particularly relevant in fields like biomedical devices, robotics, and adaptive architectures. To better understand these aspects, a Venn diagram in Figure 2 illustrates the key components of different AM techniques, including process types, materials used, and applications. Surrounding petals emphasize the main advantages and limitations associated with each technology.

However, both 3D and 4DP of polymer composites come with inherent challenges. The mechanical properties of printed parts can depending on the printing parameters, the type of polymer matrix used, and the incorporation of reinforcing fillers. In 4DP, the complexity increases further, as materials need to exhibit predictable and controllable transformations. The quality and functionality of printed parts are influenced by numerous factors, such as material selection, process control, and post-processing, making it difficult to optimize production efficiently using traditional trial-and-error methods [46,47].

**Figure 3:**
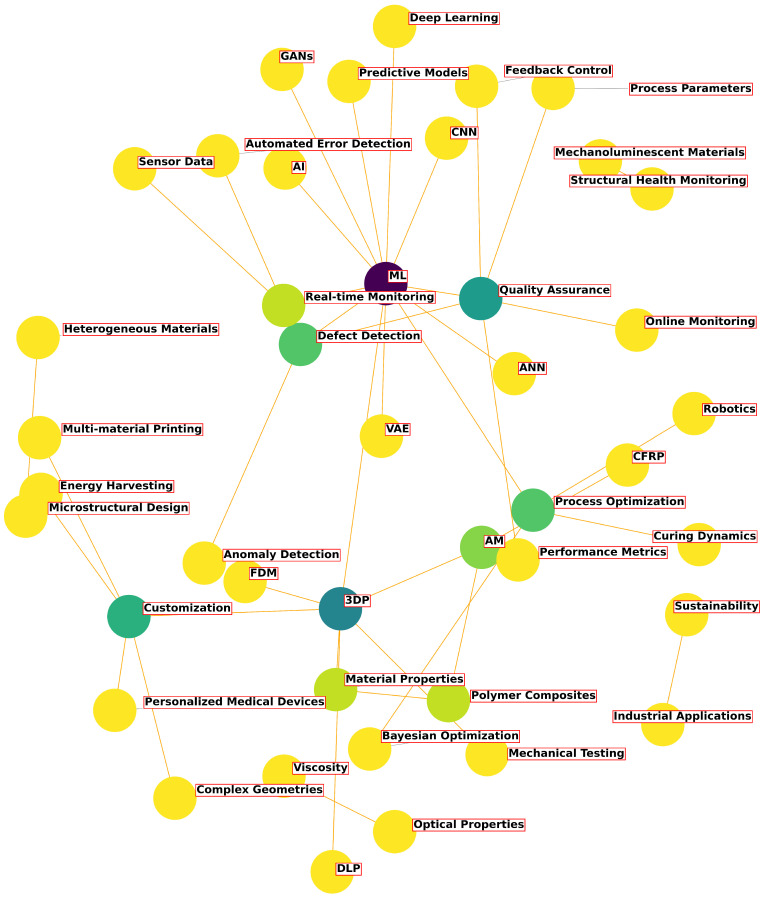
Bibliometric network visualization.

Figure 3 presents a conceptual graph that illustrates the interconnections among key concepts in this domain. The graph includes elements, such as ML methods (e.g., artificial neural networks, variational autoencoders, and generative adversarial networks), as well as their applications in quality control, defect detection, and process optimization.

This approach helps us to better understand how different components interact with each other and identifies promising directions for further research and development in the fields of ML and AM. ML has the potential to predict the performance of polymer composites in various environmental conditions and assist in real-time monitoring of the printing process, ensuring quality and consistency [48,49]. By leveraging ML, the development of smart materials and structures can become more efficient, reducing experimentation time and enhancing the overall reliability of 3D- and 4D-printed products.

### 2.1. Material Extrusion

**Figure 4:**
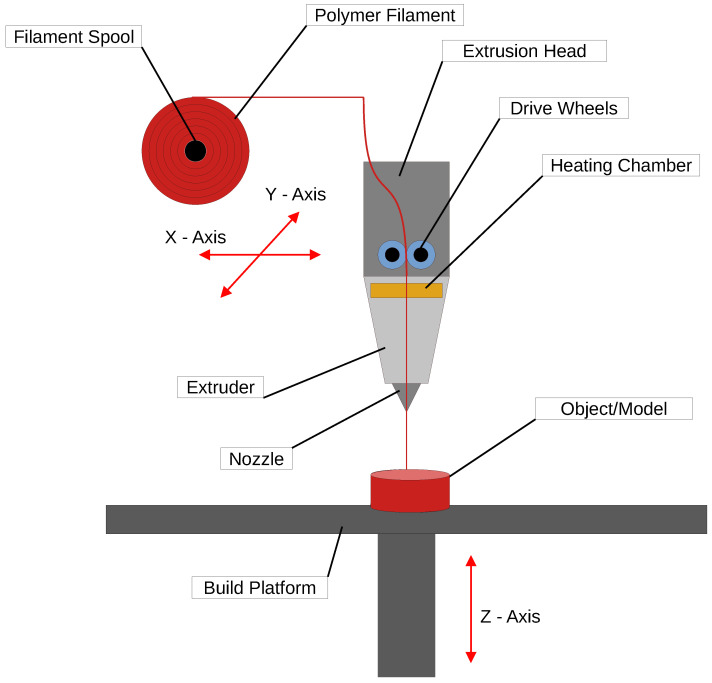
Schematic representation of FDM process.

Material extrusion, the FDM process, also known as FFF, is an AM technique in which a 3D object is constructed layer by layer using melted material [50,51,52,53,54,55,56,57,58,59]. The process begins with model preparation, where a 3D model is created using CAD (Computer-Aided Design) software. This model is then processed by slicing software, which divides the model into horizontal layers and generates instructions, or G-code, for the 3D printer. A schematic representation of the process is shown in Figure 4.

Next is the material feeding stage. The material, typically a thermoplastic filament, is fed into the printer’s extruder, which heats it until it reaches a semi-liquid state [60]. Extrusion and deposition follow, where the melted material is extruded through a nozzle onto the build platform. The print head follows a set path, depositing material layer by layer. As each layer is completed, the nozzle or build platform moves up to prepare for the next layer [61].

The object is then built layer by layer, with the material gradually forming the 3D shape. For complex geometries or overhangs, the printer can deposit a secondary, support material alongside the main material to hold these structures in place [62].

After deposition, each layer cools and solidifies, locking into the layer below. This process repeats until the entire model is complete. In the final stage, the object is removed from the platform, and any support structures are detached, either mechanically or by dissolving if they are water-soluble. Final steps may include surface treatments, such as sanding, polishing, or painting, to improve surface quality [63].

#### 2.1.1. Fused Deposition Modeling

Subramani et al. identify optimal FDM parameters for ABS components; Melentiev et al. improve adhesion in multi-material parts by combining FDM with chemical deposition and electroplating; Bahrami et al. enhance wear resistance in Fe–ABS composites using a GA-ANN optimization model; and Hajjaj compares the mechanical properties of zirconia restorations produced by FDM and CAD/CAM milling, finding FDM-printed zirconia to be mechanically inferior.

Subramani et al. [64] investigate how various FDM parameters, such as infill density, printing speed, platform, and extruder temperature, affect the mechanical properties of ABS components produced on a Creality Ender-3 3D printer [64]. Mechanical properties like tensile strength, yield strength, and elastic modulus were evaluated using a Multi-Criteria Decision-Making (MCDM) [65] approach [66]. The optimal printing settings—35% infill, 0.25 mm layer height, 40 mm/s speed, 75 °C platform, and 210 °C extruder temperature—were identified for manufacturing impellers. Additionally, field emission scanning electron microscopy (FESEM) [67] provided insights into surface defects and material behavior.

Melentiev et al. [68] explore multiprocess additive manufacturing (MPAM) to produce multi-material components, focusing on improving the adhesion strength between metal and polymer interfaces in 3D-printed parts. By combining FDM with chemical deposition and electroplating [69], the research aims to enhance the structural integrity of metalized plastic components, which typically suffer from poor adhesion. The study focused on creating a hierarchically structured surface on ABS parts through 3DP and acid etching copper adhesion [70]. The experiment involved 3DP, surface treatment, copper deposition, electroplating, and adhesion testing, offering insights for industries using MPAM for advanced electronics and multi-material devices.

FDM has limitations in wear resistance [71]. To address this, Fe particles (10%, 20%, and 30%) were added [72] to ABS to create Fe–ABS composite filaments, and parts were printed using varying filling patterns, nozzle temperatures, and layer thicknesses. Wear testing showed that Fe percentage had the greatest effect on wear reduction, followed by filling pattern, while nozzle temperature had the least impact. For optimization, a genetic algorithm–artificial neural network (GA-ANN) [73] model slightly outperformed the response surface methodology (RSM) [74], with results closely matching experimental data at a 0.25% error rate.

Hajjaj [75] compares the mechanical properties of zirconia dental restorations made using 3DP (FDM) and CAD/CAM milling, focusing on the effects of conventional versus speed sintering. A total of 60 bars were tested for flexural strength and modulus, while 40 discs were used for Vickers microhardness testing. Results showed that milled zirconia had higher flexural strength and modulus than FDM-printed zirconia. The sintering cycle did not affect flexural properties, but speed sintering the Vickers microhardness of milled zirconia did have an effect. Overall, FDM-printed zirconia was mechanically inferior to milled zirconia.

#### 2.1.2. Fused Filament Fabrication

This section summarizes recent studies on FFF. Khan reviews how process parameters affect the mechanical properties of lightweight polymers; Kariuki identifies optimal printing parameters for carbon fiber-reinforced polyamide 12; Garcia compares FFF, Metal Injection Molding, and Powder Metallurgy for 17-4 PH stainless steel, noting FFF’s superior tribocorrosion resistance; and Kalinke explores sustainable practices in FFF using renewable and recycled materials to enhance environmental sustainability.

Fused filament fabrication (FFF) is a cost-effective 3DP method for lightweight polymer structures. Key mechanical properties like flexural and impact strength are influenced by process parameters and material selection [76]. Filled polymers often perform better, and crystallinity plays a key role in the final properties. Review [76] discusses emerging trends such as topology optimization and polymer recyclability, while highlighting research gaps and proposing directions for further in FFF technology.

Kariuki et al. [77] investigate the flexural behavior of 3D-printed short carbon fiber-reinforced polyamide 12 (PA12-CF) [78] parts produced using fused filament fabrication (FFF). Using an L18 Taguchi design and Gray relational analysis, the optimal printing parameters were identified. Build orientation had the most impact on flexural properties, with a rectilinear infill pattern producing a flexural strength of 119.9 MPa and modulus of 3038 MPa, while a concentric pattern improved strength by 15.8%. This work provides valuable insights into optimizing FFF parameters for enhanced mechanical performance in carbon fiber composites.

Garcia et al. [79] compare the effects of different manufacturing methods— FFF, Metal Injection Molding (MIM), and conventional Powder Metallurgy (PM)—on the properties of 17-4 PH stainless steel. FFF and MIM both produced near-dense parts, but MIM samples showed the highest hardness. Corrosion behavior was similar for FFF and MIM, both outperforming PM. However, FFF parts exhibited superior tribocorrosion resistance, attributed to higher proportions of delta ferrite and retained austenite in their microstructure. These findings highlight the potential of FFF for producing corrosion-resistant, durable components.

Kalinke et al. [80] explore sustainable methods for enhancing the development, treatment, and applications of 3D-printed objects, particularly in FFF. The paper discusses various conductive and non-conductive filaments made from renewable biopolymers [81], bioplasticizers, and recycled materials, detailing how these choices impact material properties. They also highlight alternative strategies for sustainability, including recycling, adjusting printing parameters, and system miniaturization. These approaches aim to reduce environmental impact while producing high-quality, cost-effective 3D-printed products, aligning with Green Chemistry principles and Circular Economy concepts.

### 2.2. Stereolithography

In SLA printing for polymer composites [44,57,58,59,82,83,84,85,86,87], the process is adapted to use specialized resin blends that incorporate composite materials, such as ceramic, carbon, or glass fibers, to enhance the mechanical properties, thermal resistance, or surface finish of the printed object. This approach combines the precision and detail of SLA with the strength and functionality of composite materials, creating parts that are suitable for more demanding applications.

The process begins with model preparation in CAD software, where a 3D model is designed and then sliced into layers. The composite resin is prepared in a vat and often contains finely distributed particles (e.g., glass, ceramic, or carbon fibers) that are suspended within the photopolymer base. This composite resin is carefully formulated to maintain a uniform consistency, ensuring that particles do not settle and are evenly distributed throughout each layer of the print.

In the layer curing phase, a UV laser or projector selectively cures each layer, hardening both the photopolymer and the embedded particles simultaneously. The UV laser follows a precise path to solidify each layer, bonding the particles into a matrix that enhances the overall strength and durability of the printed part [88]. Between each layer, the platform moves incrementally to allow the next thin layer of resin to coat the surface [89].

The layer-by-layer bonding process creates a composite structure, embedding the particles within the cured photopolymer matrix [90]. Support structures are added automatically by the slicing software when needed, especially for overhangs or complex geometries, and are printed in the same composite material [91].

Once the print is completed, post-processing begins. The part is removed from the resin vat, cleaned of any excess resin, and may undergo an additional UV curing process to fully harden the composite [92]. Support structures are then removed, and the object may be further processed through sanding, polishing, or coating, depending on the application’s requirements [93].

**Figure 5:**
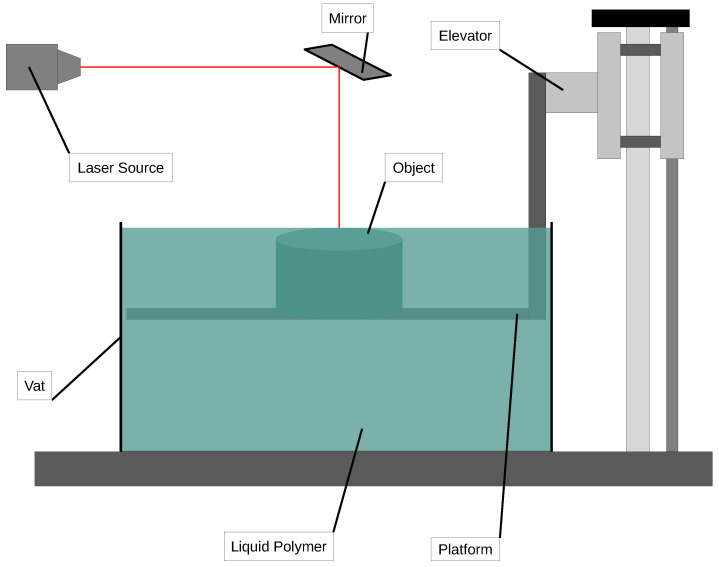
Schematic representation of SLA process.

SLA printing with polymer composites enables the production of parts with improved mechanical properties [94], thermal stability [95], and surface quality [96], making it ideal for engineering prototypes, end-use parts, and high-performance applications in industries such as aerospace, automotive, and medical devices. This technique expands the scope of SLA by providing a balance between high resolution and enhanced material strength, tailored to meet the specific demands of advanced manufacturing. A schematic representation of the SLA process is shown in Figure 5.

Hydrogel-based electronics are promising for wearable devices but face challenges like low conductivity and stretchability. Sun et al. [97] present a projection SLA 3DP method to create high-conductive, flexible hydrogel antennas for wireless sensing [98]. The photocurable silver-based hydrogel forms conductive pathways after partial dehydration, achieving a conductivity of 387 S cm^−1^. Sealed circuits maintain stable resistance under 100% strain for 30 days, with added features like stretchability and shape memory. Custom flexible RFID tags were created, enabling accurate eye movement tracking and passive wireless sensing.

Zhou et al. [99] examine Stereolithography Additive Manufacturing (SLAM) for producing advanced ceramic objects with complex geometries [100], highlighting its resolution and surface quality. It addresses the challenges in achieving the desired performance due to the necessity of thermal debinding (TD) [99] to remove binders, which can lead to defects and prolonged processing times. Key topics covered include the impact of raw materials on photocurable ceramic suspensions, the mechanisms and characterization methods of the TD process, and strategies for designing effective TD profiles. The review concludes with insights into the challenges and future directions for TD in ceramic SLAM, providing a foundational understanding for optimizing TD processes in research and industry.

Kulkarni et al. [101] investigate the use of SLA to print polymer nanocomposite samples of stimuli-responsive spin crossover (SCO) materials with resins DS3000 and PEGDA-250. The analysis showed that incorporating SCO particles improved mechanical properties, with transformation strains of 1.2–1.5% at high loads (13–15 vol.%), enabling thermal expansion peaks. Two SCO complexes were synthesized and characterized, demonstrating their suitability for actuator applications due to favorable spin transition properties. The findings emphasize the importance of effective particle dispersion for optimal performance in SLA-printed composites.

Pharmaceutical 3DP [102,103,104,105,106,107,108,109,110,111] is advancing rapidly, offering the potential for highly personalized medicine. SLA is a particularly promising technology due to its high resolution and compatibility with heat-sensitive drugs. However, the lack of specialized excipients for pharmaceutical SLA limits material options. Curti et al. [103] investigate how formulation factors—such as photoinitiator concentration, polymer size, and liquid filler type—affect the print quality of SLA 3D-printed medicines. By screening 156 photopolymer formulations, it highlights how these factors influence print outcomes, providing valuable insights for future development of personalized 3D-printed pharmaceuticals.

### 2.3. Selective Laser Sintering

**Figure 6:**
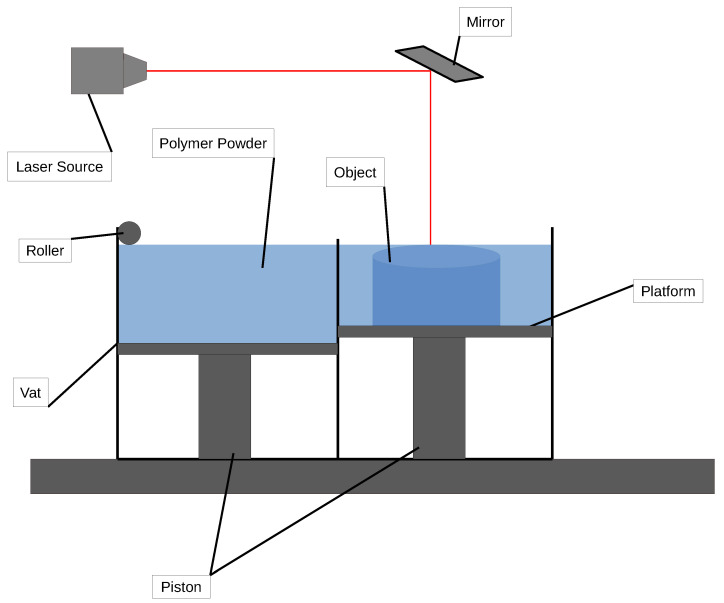
Schematic representation of SLS process.

In SLS for polymer composites printing [112,113,114,115,116,117,118,119,120,121], a powdered composite material—typically a blend of a polymer base like nylon with reinforcing particles such as carbon fiber, glass beads, or ceramic—is used to create strong, high-performance parts. This process leverages the strength and durability of composite materials within the flexibility of polymer-based 3DP, ideal for demanding applications in industries such as aerospace, automotive, and consumer goods. A schematic representation of the SLS process is shown in Figure 6.

The process starts with model preparation using CAD software, where a 3D model is designed and then processed through slicing software to divide the model into thin, horizontal layers. These layers are used to generate precise instructions for the SLS printer.

In the powder preparation stage, the composite powder is loaded into the build chamber [122]. This powder must be well-mixed to ensure an even distribution of reinforcement particles, like carbon or glass fibers, throughout the polymer base. A powder bed is formed, and the printer’s roller or recoater evenly spreads a thin layer of the composite powder across the build platform [123].

During layer sintering, a laser selectively fuses areas of the powder bed, following the contours of the sliced model layer [124]. The laser heats the polymer particles to their melting point, allowing them to fuse together while also bonding the embedded reinforcement particles within the polymer matrix. Once a layer is completed, the platform lowers slightly, and a new layer of composite powder is spread over the previous one. This layer-by-layer sintering process continues until the entire object is formed, with each new layer bonding to the one beneath it. Because of the self-supporting nature of the powder bed, support structures are typically not required, allowing for more complex geometries without additional material waste.

After printing, the object is surrounded by unsintered powder, which acts as a support and is carefully removed in the post-processing phase. The remaining loose powder is brushed or blown off, often followed by bead-blasting or compressed air cleaning to reveal the printed part. Additional finishing steps such as sanding or coating can further enhance the part’s surface quality and durability [125].

SLS printing with polymer composites produces parts with superior mechanical strength, stiffness, and thermal stability compared with traditional polymers, due to the reinforcing particles integrated within the polymer matrix. This method is well-suited for functional prototypes, tooling, and end-use parts that require the combined benefits of both high-performance polymers and reinforcement materials.

Song et al. [126] highlight SLS’s role in medical engineering for producing complex biomedical products, particularly implants and prosthetics using biocompatible materials; Azam et al. focus on SLS processing of polymer materials, emphasizing innovations in piezoresistive strain-sensing and the process–structure–property relationships; Han et al. develop a method for creating carbon nanotube-anchored α-ZrP nanohybrids to enhance polyamide 12 composites, resulting in significant improvements in mechanical properties and functional characteristics; and Zhang et al. investigate the effects of process parameters on carbon fiber-reinforced PEEK composites, achieving notable strength and modulus enhancements for industrial applications.

SLS has been particularly successful in creating electrically conductive polymer composites (ECPCs) by forming a segregated filler network along powder boundaries. Azam et al. [127] focus on SLS processing of polymer materials, highlighting the consolidation mechanisms, process parameters, and innovations in piezoresistive strain-sensing materials and self-sensing structures. They also explore the intricate process–structure–property relationships in SLS-printed polymer composites.

Han et al. [128] introduce a simple method to synthesize a carbon nanotube (CNT)-anchored α-ZrP nanohybrid (CNT@α-ZrP) for enhancing polyamide 12 (PA12) composites using ball-milling followed by SLS. CNTs serve dual functions: providing black coloration for efficient heat absorption and reinforcing the PA12 matrix. The α-ZrP nanosheets primarily enhance the mechanical and functional properties of PA12 composites. The resulting PA12/CNT@α-ZrP composites show improvements in Young’s modulus (98.9%), tensile strength (33.1%), and impact strength (34.6%), along with better wear resistance, flame retardancy, and reduced smoke production. This method offers an industrial approach to producing robust and functional SLS-based structures.

Carbon fiber-reinforced PEEK (CF/PEEK) composites fabricated via SLS offer excellent mechanical properties and are highly promising for advanced applications. Zhang et al. [120] investigate the impact of process parameters—such as laser power, layer thickness, paving speed, and carbon fiber content—on the microstructure and performance of CF/PEEK composites. Key findings include achieving a failure strength of 117 MPa with a layer thickness of 0.08 mm and an optimal fiber weight fraction of 15%. The highest elastic modulus reached 8400 MPa, surpassing previous works. The study also reveals nonlinear relationships between paving speed and strength, with longer carbon fibers improving strength. The research provides insights into optimizing SLS-CF/PEEK composites for industrial applications.

### 2.4. Digital Light Processing

**Figure 7:**
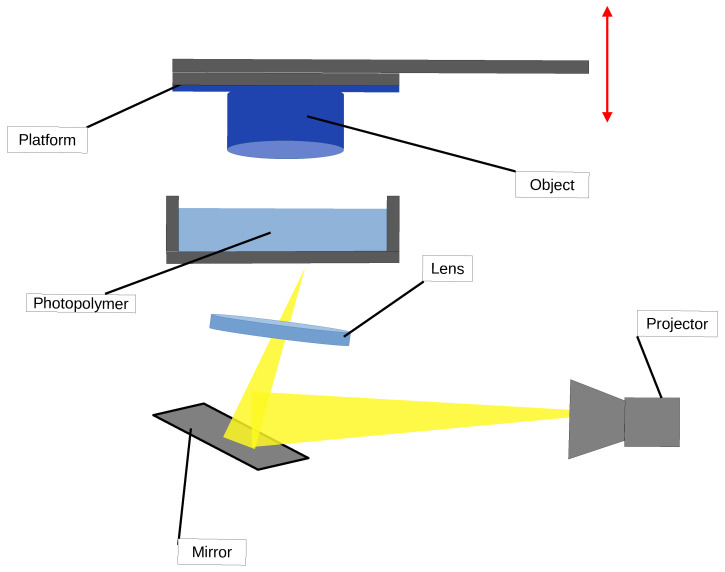
Schematic representation of DLP process.

In DLP printing for polymer composites [121,129,130,131,132,133,134,135,136,137], the process utilizes a high-resolution digital projector to cure photopolymer resins that are often blended with composite materials, such as ceramic, carbon fiber, or glass particles. This combination allows for the creation of highly detailed parts with enhanced mechanical properties, making DLP a suitable choice for various advanced manufacturing applications. A schematic representation of the DLP process is shown in Figure 7.

The process begins with model preparation in CAD software, where a 3D model is designed and optimized for printing. The model is then sliced into thin layers by slicing software, which generates the necessary instructions for the DLP printer.

In the resin preparation phase, a vat is filled with liquid photopolymer resin mixed with composite materials. The resin is specially formulated to ensure uniform dispersion of the composite particles, allowing for consistent curing and material properties throughout the printed part.

During the layer curing phase, the DLP printer uses a digital light projector to expose the surface of the resin to UV light. The projector displays a complete layer of the model at once, curing the resin in a pattern that corresponds to the sliced model [138]. This process allows for rapid curing of an entire layer simultaneously, significantly speeding up the printing process compared with traditional layer-by-layer methods.

Once a layer is cured, the build platform moves upward (or the resin vat moves downward) to allow fresh resin to flow over the cured layer, preparing for the next layer. The layer-by-layer construction continues until the entire object is complete, with each layer bonding to the one beneath it.

For complex geometries, support structures are often generated by the slicing software to prevent deformation during printing. These supports are printed using the same composite resin and can be easily removed after the printing process is finished.

After the printing is complete, the part undergoes post-processing. It is removed from the resin vat and cleaned of any excess uncured resin, typically using isopropyl alcohol. The part may then be subjected to further UV curing to ensure complete hardening. Any support structures are removed, and final finishing processes, such as sanding or coating, can be applied to achieve the desired surface quality.

DLP printing with polymer composites offers several advantages, including high resolution and smooth surface finishes, while also enhancing the mechanical properties of the printed parts. This technology is particularly well-suited for applications in industries such as dental and medical devices, jewelry, and high-performance engineering components, where precision and material strength are paramount.

Melentiev et al. [139] present lithography metal additive manufacturing (LMAM) for high-resolution metal parts with excellent density and tensile strength; Guo et al. enhance photosensitive resin with multi-walled carbon nanotubes, improving mechanical properties; Senthooran et al. incorporate mica into DLP-printed samples, significantly increasing tensile and flexural strength; and Wang et al. create a flexible multistage honeycomb absorber from carbonyl iron and MWCNTs, demonstrating exceptional electromagnetic wave absorption.

DLP is employed in high-resolution AM, enabling the 3DP of complex metallic parts with micrometer precision. Melentiev et al. [139] present lithography metal additive manufacturing (LMAM), a method that utilizes DLP with a photosensitive resin filled with metal powder. The process yields intricate structures with a spatial resolution of 35 μm and surface roughness of 1–2 μm without support structures. Sintered stainless steel parts exhibit 99.3% density and 93% tensile strength relative to annealed 316 L steel. LMAM is ideal for fabricating small, precise devices in fields such as biomedicine, microheat exchangers, and pharmaceutical engineering.

Guo et al. [137] explores the potential of multi-walled carbon nanotubes (MWCNTs) to enhance the structural, mechanical, and electrical properties of materials through AM, specifically focusing on DLP techniques. Despite the growing interest in MWCNT-reinforced composites, there is limited research on their integration into photosensitive resin (PR) systems using DLP, particularly concerning the distribution patterns of MWCNTs. This investigation fabricated MWCNTs-reinforced PR (MWCNTs-PR) and examined how varying MWCNT content affects the microstructure and mechanical properties of the composite. Findings indicate that adding 0.05 wt% MWCNTs enhances the elastic modulus by 25% and the bending strength by 2% compared with pure PR. To achieve a more uniform MWCNT distribution, a combination of ultrasonic treatment and mechanical stirring was employed. The study further developed a multi-material layered 3DP structure, demonstrating that the 10001 structure achieved the highest bending modulus, outperforming the control group by 14.9%. Finally, finite element analysis was utilized to validate the enhanced bending resistance mechanism attributed to the MWCNTs in the PR.

Senthooran et al. [133] explore the enhancement of mechanical and thermal properties in 3D-printed samples using DLP by incorporating mica as an inorganic filler at 5%, 10%, and 15% concentrations, along with a KH570 silane coupling agent for better dispersion. The results show improvements: tensile strength increased by 85% and flexural strength by 132% with mica addition. Thermogravimetric analysis (TGA) and scanning electron microscopy (SEM) were used for thermal and morphological evaluations. The findings highlight advancements in AM technology through DLP techniques.

Research on wideband electromagnetic (EM) absorbers in the 75–110 GHz range is limited, hindering millimeter-wave technology advancements. Wang et al. [140] introduce a novel flexible multistage honeycomb structure absorber (FMHSA) made from carbonyl iron (CIP), multi-walled carbon nanotubes (MWCNTs), and flexible photopolymer resin (FPR), fabricated via DLP 3DP. The FMHSA achieves exceptional EM wave absorption with a bandwidth of 35 GHz at a 150° bending angle and a minimum reflection loss of −37.04 dB. Its notable properties include flexibility, recoverability, and lightweight design, paving the way for improved wearable absorbers.

### 2.5. Jetting 3D Technologies

Jetting 3D technologies include MJF and PJP, both of which can utilize polymer composites to create high-performance parts with enhanced mechanical properties.

**Figure 8:**
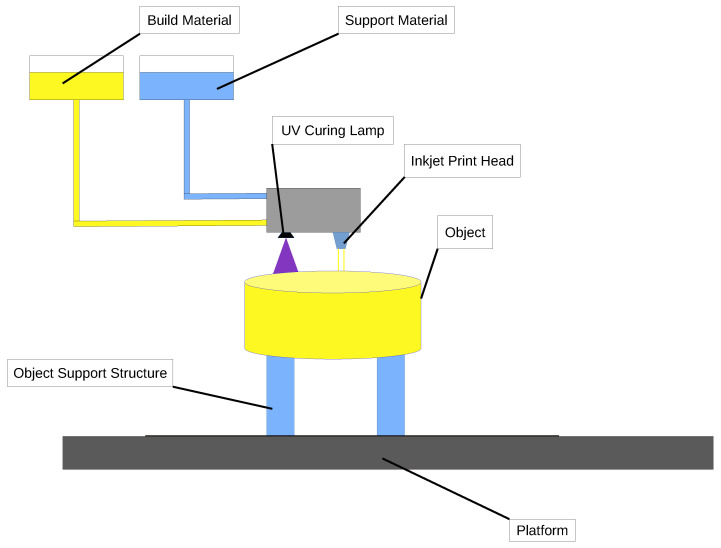
Schematic representation of MJM process.

In Multi Jet Fusion [141,142,143,144,145,146,147,148,149,150], a layer of polymer composite powder, often a blend of nylon and reinforcing materials like carbon fiber or glass beads, is spread across the build platform. Inkjet print heads selectively apply a fusing agent to specific areas of the powder bed, allowing for controlled heating and fusion when exposed to infrared light. This layer-by-layer process continues until the part is fully formed, with each layer bonding to the previous one. After cooling, excess un-fused powder is removed and can be recycled. MJF produces parts that exhibit superior strength and durability, making it ideal for functional prototypes and end-use applications in industries such as aerospace and automotive. A schematic representation of the MJM process is shown in Figure 8.

PolyJet printing [151,152,153,154,155,156,157,158,159,160] utilizes a different approach by jetting ultra-thin layers of liquid photopolymer resin, often enhanced with composite materials. The print heads spray the resin, which can include reinforcing particles, and immediately cure it with UV light. This method allows for the incorporation of various materials in a single print job, enabling the creation of parts with tailored mechanical properties and surface finishes. After printing, support structures are easily removed, resulting in high-resolution parts. PolyJet is particularly well-suited for applications requiring intricate details and multi-material capabilities, such as dental devices and intricate consumer products.

#### 2.5.1. Multi Jet Fusion

Alomarah et al. find that MJF outperforms FFF in producing stronger auxetic structures; Tan et al. develop a framework showing how fiber weight affects porosity in fiber-reinforced composites; Kafi et al. explore the impact of build height and orientation on the mechanical properties of MJF-printed polypropylene; and Conway et al. assess the geometric accuracy of MJF surgical guides, achieving precise distortion predictions using machine learning.

MJF and FFF are explored for fabricating a hybrid auxetic structure in AM. Alomarah et al. [143] find that MJF produces robust specimens with high dimensional accuracy, while FFF suffers from large pores in connecting areas, indicating lower print quality. MJF specimens exhibit plateau stress with high peaks when compressed along the *Y*-axis, whereas FFF specimens display a smooth plateau stress. MJF specimens achieve the highest specific energy absorption (SEA) at 2.1 and 2.5 J g^−1^, exceeding the 0.495 and 0.480 J g^−1^ of FFF specimens. Additionally, the auxetic features (negative Poisson’s ratio) remain unaffected by the manufacturing methods. This research underscores the influence of fabrication techniques on the mechanical properties and energy absorption capabilities of cellular materials.

AM of fiber-reinforced polymer composites is gaining attention for its ability to create lightweight, functional products. However, pore defects remain a concern, necessitating a better understanding of pore formation. Tan et al. [117] present a powder-scale multi-physics framework to simulate the printing process of fiber-reinforced polymer composites in powder bed fusion in MJF. The framework incorporates various phenomena, including particle flow dynamics, laser–particle interaction, heat transfer, and multiphase fluid flow. The melt depths of glass fiber-reinforced polyamide 12 parts fabricated via selective laser sintering were measured to validate the model. Results indicate that increasing the fiber weight fraction leads to a lower densification rate, larger porosity, and reduced pore sphericity in the composites.

Kafi et al. [142] validate the absorption phenomena in MJF-printed polypropylene (PP) using Laser Flash and Corrected Porosity methods. It investigates how build height and orientation affect tensile properties, crystallinity, porosity, and thermophysical attributes in MJF-printed PP coupons. Results indicate that crystallinity and tensile performance are consistent across orientations, but Z-oriented samples exhibit 35% lower strain and increased porosity compared with XY samples. Micro-CT scans revealed that horizontal positioning improved contrast for porosity analysis. A correlation was established between Laser Flash half-time and porosity when corrections were applied, indicating that lower absorption occurs in less dense Z samples. The findings highlight the importance of accurately determining porosity to understand absorption in MJF-printed PP, offering insights into predicting mechanical properties and enhancing the overall quality of MJF-produced parts.

Conway et al. [161] examine the repeatability and geometric accuracy of AM surgical guides for personalized knee surgery. A total of 258 unique guide designs were created, and 2100 parts were produced using MJF AM. An automated measurement technique gathered 8400 individual feature dimensions, revealing standard deviations in feature size ranging from 0.076 to 0.173 mm and consistent deviations from target dimensions of −0.308 to 0.017 mm. ML models were developed to predict these geometric distortions, achieving accuracy within 0.033 to 0.075 mm, allowing for effective predictions across various part sizes.

#### 2.5.2. PolyJet Printing

This section highlights advancements in PolyJet printing. Azpiazu et al. assess how thermocycling and surface finishing impact the strength and hardness of dental prostheses, finding certain finishes yield better performance. Krause et al. examine material choice and print orientation effects on microfluidic channel accuracy, noting optimal results for wider channels. Aberdeen et al. explore the interface design and mechanical failure of bi-material coupons, emphasizing the need for further research on geometric designs to strengthen material interfaces.

PolyJet 3DP is an advanced AM technology that deposits photopolymeric materials in micron-sized droplets, curing them with ultraviolet (UV) light. It excels in creating complex, multi-material structures with exceptional precision, achieving layer thicknesses as fine as 16 microns [162]. Its versatility allows for a wide range of materials, including rigid, flexible, and transparent options, enabling the production of components with tailored mechanical and optical properties. While widely used in industries like aerospace and healthcare, challenges remain in material performance and print optimization, necessitating ongoing research to enhance interfacial bonding and mechanical properties.

Azpiazu et al. [163] evaluate the effects of thermocycling and different surface finishing protocols on the flexural strength and surface hardness of a novel photopolymer designed for monolithic polychromatic dental prostheses made via PolyJet 3DP. A total of 90 specimens were divided into three groups based on finishing protocols: Pumice + Moldent, Pumice + Optiglaze, and Polycril + Moldent. Results showed that thermocycling reduced the flexural strength across all groups, with the Optiglaze group demonstrating the highest strength after thermocycling. The analysis also revealed an interaction between thermocycling and finishing protocols concerning surface hardness, with the Optiglaze group exhibiting the highest hardness values.

Krause et al. [164] investigate the impact of materials and print orientations on the 3DP of microfluidic channels as negative features using PolyJet technology and the Stratasys Objet500 printer. Two sets of chips, each containing channel pairs made from a high-contrast reference material and a sacrificial material embedded in clear photopolymer resin, were printed. The planned channel widths ranged from 64 to 992 μm, and the channels were printed either parallel or perpendicular to the jetting head’s movement. The findings indicate that reproducibility and accuracy were optimal for channels with a width of 600 μm or greater, with the best channel morphology achieved when the printer head moved parallel to the channel’s longitudinal axis.

Aberdeen et al. [165] explore the interface design and mechanical failure dynamics of PolyJet-printed bi-material coupons using material jetting technology, specifically PolyJet 3DP. By investigating various geometric designs and conducting uniaxial tensile tests on samples printed with a Stratasys Objet500 Connex3 printer, the results reveal that increasing the surface contact area between distinct materials does not necessarily enhance interface strength. The findings highlight the need for further research into multi-material geometric designs and their impact on interface integrity, particularly as interest in PolyJet printing grows in applications like robotics and fluidic circuitry.

### 2.6. Direct Ink Writing

DIW is AM technique that focuses on extruding viscoelastic inks [166,167,168,169,170,171,172,173,174,175], which can be formulated from polymer composites, to create complex geometries with enhanced material properties. This method is particularly effective for producing parts with tailored mechanical characteristics and functionality, suitable for a variety of applications.

The DIW process begins with ink formulation, where a composite ink is created by blending a polymer matrix with reinforcing materials, such as carbon fibers, glass fibers, or ceramic particles [176]. This ink must possess the right viscosity and flow properties to be extruded through a nozzle while maintaining shape fidelity after deposition.

During the printing phase, a syringe or nozzle extrudes the composite ink layer by layer onto a build platform. The printer’s movement is controlled by a computer program that follows a pre-defined path, allowing for precise placement of material [177]. As each layer is deposited, it retains its shape due to the viscoelastic properties of the ink, enabling the creation of complex structures, including overhangs and intricate designs.

**Figure 9:**
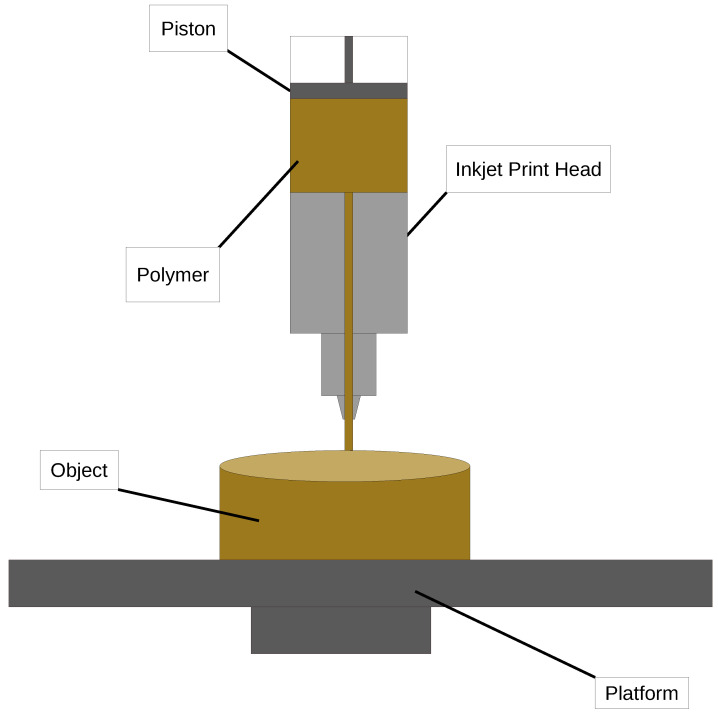
Schematic representation of DIW process.

Post-processing is often required after printing, which may involve curing the printed part through heat or UV light, depending on the type of polymer used. This curing process solidifies the polymer matrix, enhancing the mechanical strength and durability of the final part [88,178]. Additionally, support structures may be incorporated or added during the printing process to ensure stability for more complex geometries. A schematic representation of the DIW process is shown in Figure 9.

DIW enables precise layer-by-layer deposition of functional materials through a positive displacement dispensing system [179]. It excels in printing on flexible substrates, such as polyethylene terephthalate (PET), due to its adaptability to various material viscosities and the importance of controlling process parameters like air pressure and feed rate for achieving high-resolution patterns. One of DIW’s advantages is its ability to print conductive inks, crucial for developing sensors and electronic components, and its capability for multi-material printing, allowing for the creation of complex, multifunctional structures. This makes DIW suitable for fabricating stretchable and bendable sensors that can monitor mechanical deformations by varying electrical resistance, while the deposited patterns are typically cured to enhance structural integrity and performance in wearable technologies [180].

The emergence of 3DP technology in the 1980s has facilitated the creation of patient-specific products with precise shapes and complexities. Among various techniques, DIW is favored for its affordability, ease of use, and scalability, although the limited variety of printing inks hampers its commercial potential. Injectable hydrogels, known for their quick gelling behavior and shape fidelity, have emerged as promising alternatives for printing inks, made from natural or synthetic polymers to achieve desired properties. Bhardwaj et al. [173] highlight recent advancements in hydrogel inks and their physicochemical aspects for engineered biostructures, and discuss the future prospects and challenges of 4DP in hydrogel-based 3DP applications in healthcare.

DIW advances hydrogel fabrication by enabling precise layer-by-layer deposition of hydrogel inks to create complex three-dimensional structures with tailored properties. Baniasadi et al. [181] explore the diverse applications of DIW in areas such as tissue engineering, soft robotics, and wearable devices, while also examining the various printing techniques and the underlying principles of DIW, including rheological properties and printing parameters. Additionally, they highlight the range of natural and synthetic hydrogel materials used in this process and discuss the latest biomedical applications, particularly in tissue engineering, wound dressings, and drug delivery systems, while outlining future research directions and potential innovations in hydrogel-based manufacturing.

DIW offers a flexible and resource-efficient method for prototyping functional materials and devices with complex geometries. Van et al. [182] focus on the use of graphene nanoplatelets (GNPs) as conductive fillers in printed electronics, addressing the challenges posed by non-spherical colloids that risk nozzle clogging. A workflow was developed to optimize ink rheology and printing parameters, enabling the successful production of filaments ranging from <100 to 1200 μm in width and 30 to 300 μm in height, with conductivities suitable for sensors and electrodes. The predictive models created from this research facilitate high-resolution DIW of platelet-based inks, promoting integrated material and process development for applications in wearable electronics, sensors, RF passives, energy materials, and tissue engineering.

### 2.7. 4D Printing

This section covers advancements in 4DP, which adds the dimension of time to traditional AM by using materials that change shape in response to external stimuli. Khalid et al. emphasize the potential of shape memory polymers (SMPs) that react to stimuli like heat and humidity for use in various fields. However, challenges like mechanical limitations remain. Qiu et al. explore the benefits of fiber-reinforced polymer composites (FRPCs) in 4DP, enhancing mechanical performance. Yan et al. review SMP composites in 4DP, summarizing advancements and discussing future prospects in biomedical application

Current FDM technology enables the use of multiple polymer filaments, paving the way for complex, responsive structures [63]. Four-dimensional printing represents an advancement beyond traditional 3DP by incorporating the dimension of time. This innovation is made possible through the development of intelligent materials that change shape in response to external stimul. The most promising applications of 4DP are in the creation of smart textiles, which can act as actuators and sensors, allowing for bio-inspired designs. Key areas of potential include smart clothing for extreme environments, auxiliary prosthetics, and orthotic devices that aid muscle recovery.

In recent years, there has been growing interest in AM shape memory polymers (SMPs) and their multifunctional composites, particularly in the realm of four-dimensional (4D) printing, which utilizes time-responsive programmable materials. These stimuli-responsive polymers can return to their original shapes from programmed temporary forms upon exposure to external stimuli such as heat, light, or humidity. The integration of 4DP with shape memory polymer composites (SMPCs) opens up a wide range of engineering applications [183,184,185], including in automotive, soft robotics, biomedical devices, and wearable electronics. Khalid et al. [186] highlight key 4DP technologies and their functionalities, discuss future opportunities in preprogramming, multi-material printing, and sustainability, and provide illustrative examples of applications, aiming to foster advancements and innovations in the field of 4DP.

Four-dimensional printing technology has gained considerable attention for its capability to reshape 3D-printed structures in response to external stimuli over time. However, challenges such as inadequate mechanical properties, low energy output, and limited design flexibility persist in the 4DP of pure polymers. The advent of fiber-reinforced polymer composites 4DP (FRPCs-4DP) offers promising solutions to these challenges by enhancing mechanical performance and improving actuation capabilities. Qiu et al. [187] explore recent advances in FRPCs-4DP, emphasizing the role of fibers, material compositions, AM techniques, and design strategies, while also outlining the key challenges and future trends for practical applications in this emerging field.

Yan et al. [33] review SMP composites and 4DP technologies, highlighting unique 4D-printed structures and summarizing recent research progress in various fields, particularly biomedical applications. They also discuss the challenges and future prospects for 4D-printed SMPs, serving as a reference for ongoing research and practical applications.

Table 3 provides an overview of the advantages and disadvantages of 3DP methods discussed in the recent literature.

## 3. Properties of Polymer Composites in AM Technologies

**Figure 10:**
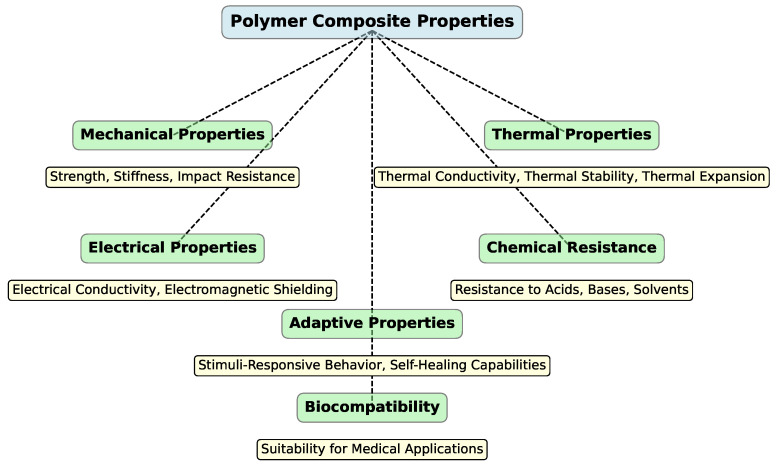
Diagram illustrating key properties of polymer composites in AM.

Polymer composites exhibit unique property combinations. These materials integrate a polymer matrix with reinforcing elements (fibers, particles, nanomaterials), achieving improvements in mechanical, thermal, electrical, and other performance characteristics. Such enhancements are important for applications in aerospace, automotive industries, medicine, and electronics. However, utilizing polymer composites in AM requires careful consideration of their structure, properties, and processing characteristics. Figure 10 illustrates the primary properties of polymer composites used in AM technologies.

### 3.1. Mechanical Properties

One of the key advantages of polymer composites is their ability to provide high mechanical performance with relatively low weight. This is achieved by incorporating reinforcing materials into the polymer matrix, such as carbon or glass fibers. Fiber-reinforced polymer composites enhance strength and stiffness compared with pure polymers. For instance, adding carbon fibers increases tensile strength, making these materials promising for structural components in aerospace and automotive applications, where high strength-to-weight ratios are essential [188]. Additionally, polymer composites exhibit excellent energy absorption characteristics, which makes them resilient to impact loads [189]. In 3DP, impact resistance is particularly important for creating prototypes and end-use parts that are subjected to dynamic stresses. Incorporating high-modulus reinforcing materials increases the stiffness of composites, which means that stiffer parts can be printed without the need for complex metalworking techniques [190]. A key challenge in AM is achieving uniform distribution of reinforcing materials to prevent defects such as delamination or weak zones that can negatively affect the mechanical properties of the printed parts [191].

### 3.2. Thermal Properties

Thermal resistance is another parameter for polymer composites in AM, especially for high-temperature applications. Adding fillers such as carbon nanotubes, graphene, or metallic particles can improve the thermal conductivity of polymer composites [192]. Aged PLA filaments filled with graphene and carbon nanotubes exhibit improved crystallinity, thermal stability [193], and electrical conductivity but reduced strength and toughness, with annealing treatments enhancing their properties based on the filler type and annealing temperature [194,195]. This is particularly relevant for creating heat-dissipating components, such as heat sinks or electronic housings. By incorporating high-temperature polymers or heat-resistant fillers, composites can withstand higher temperatures compared with standard polymers [196,197]. This makes them suitable for use in high-temperature environments, such as engine components or aerospace structures [87]. Furthermore, composites with low thermal expansion coefficients are more stable against temperature-induced dimensional changes [198], which is important for printing large or precise parts where temperature fluctuations can affect the final dimensions and geometry [199].

### 3.3. Electrical Properties

Modern polymer composites used in AM can exhibit notable electrical characteristics [200,201]. Electrically conductive polymer composites, combining polymers with metal-like electronic properties, show great potential in additive manufacturing for creating complex designs and rapid production [202], with advancements in various 3DP methods enabling breakthroughs in flexible electronics, energy storage, and other applications [203]. This enables the creation of 3D-printed parts with high electrical conductivity, suitable for sensors [204,205], antennas [206,207], and other functional devices [153,208]. Conductive polymer composites (CPCs) can also provide effective electromagnetic shielding, making them useful for printing enclosures for sensitive electronics [209]. For instance, Maleki et al. [210] created CPCs using material extrusion additive manufacturing by mixing multi-wall carbon nanotubes (MWCNTs) with ABS, resulting in 3D-printed specimens with 26 times higher electrical conductivity, improved electromagnetic interference shielding, and enhanced tensile strength and modulus, though nozzle wear occurred due to the abrasive nature of CNTs [211]. Achieving high electrical properties in AM requires ensuring uniform dispersion of conductive fillers in the matrix and preventing agglomeration, which can adversely affect both electrical properties and mechanical strength [212,213,214].

### 3.4. Adaptive Properties and 4DP

Polymer composites can also possess adaptive properties, which are particularly relevant for 4DP, where materials can change their properties or shape in response to external stimuli [215,216]. Polymer composites with shape memory properties can alter their structure or geometry in response to stimuli such as heat, moisture, or other environmental factors. This capability is utilized in 4DP to create products that can change shape over time or in response to operational conditions [217]. Additionally, some polymer composites can include elements that allow the material to self-heal after damage. In AM, such composites can be used to create parts capable of repairing themselves, thus extending their service life [218,219]. Four-dimensional printing with these composites opens new possibilities for creating adaptive and intelligent materials that can be applied in fields such as medicine, robotics, construction, and other advanced areas.

## 4. Application of ML in 3DP of Polymer Composites

**Figure 11:**
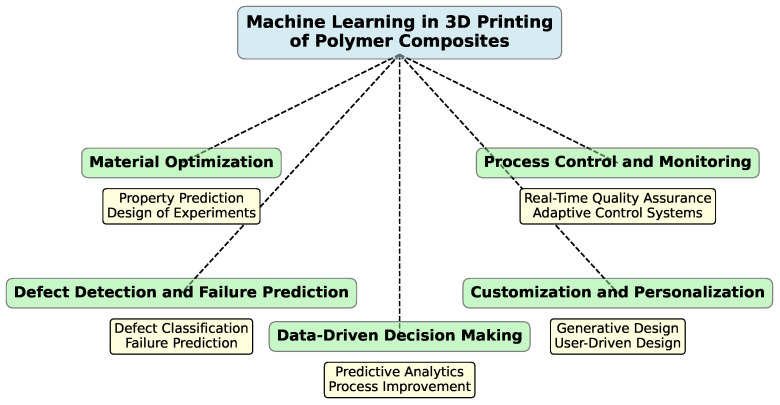
Applications of ML in 3DP of polymer composites.

ML has emerged as a transformative technology in the field of AM. By leveraging data-driven approaches, ML techniques offer improvements in various aspects of the printing process, from material optimization to process control and defect detection. This section explores the key applications of ML in 3DP of polymer composites and highlights how these innovations are reshaping the industry. Figure 11 visually organizes the various applications of ML in AM.

### 4.1. Properties Prediction

One of the primary applications of ML in 3DP of polymer composites is material optimization. Traditional methods of developing and refining polymer composite materials can be time-consuming and costly, often requiring extensive experimental trials. ML algorithms, particularly those involving supervised learning and optimization techniques, can expedite this process by analyzing large datasets of material properties and performance metrics.

FDM has enabled personalized drug-loaded formulations tailored to patient needs [220]. However, optimizing fabrication parameters is traditionally time-consuming and requires expert input. To address this, M3DISEEN, a web-based software, was developed [221], utilizing AI and ML techniques (MLTs) to enhance FDM 3DP, including filament production via hot melt extrusion (HME). AI models predict key parameters with high accuracy [222,223], streamlining 3DP for drug development. M3DISEEN is publicly available.

Three-dimensional printing in healthcare enables personalized medicines and devices but is hindered by the lengthy trial-and-error formulation process [224]. Ong et al. [225] combine in-house and literature-mined data on hot melt extrusion (HME) and fuse deposition modeling (FDM) formulations to create a balanced dataset of 1594 formulations, enhancing ML predictive performance. Optimized ML models achieved 84% accuracy in predicting printability and mechanical characteristics, with mean absolute errors of 5.5 °C and 8.4 °C for processing temperatures in HME and FDM, respectively. These models are integrated into the M3DISEEN web application, streamlining the formulation development workflow in pharmaceutical 3DP and improving research throughput [63].

Porous designs, like truss- and sheet-based lattices, offer versatility, but evaluating numerous material-lattice combinations is impractical. Peloquin et al. [226] present a framework for rapidly predicting the mechanical properties of 3D-printed gyroid lattices using base material and porosity data. A kernel ridge regression ML [227] model was trained on experimental data, achieving similar accuracy to numerical simulations but with reduced computation time, advancing ML-driven mechanical property prediction.

AM faces adoption challenges due to inconsistent product properties. Khusheef et al. [228] introduce a novel predictive method using in-process sensing to improve part property prediction in fused deposition modeling (FDM). By integrating Inertial Measurement Unit (IMU) sensors, a thermal camera, and machine settings, the study focuses on predicting key mechanical properties like tensile strength and surface roughness. Utilizing hybrid deep learning models (CNN-LSTM), the best model achieved 99% accuracy in predicting tensile strength. These results highlight the potential of sensor data and advanced modeling to enhance AM reliability and broader industry adoption.

AM of carbon fiber (CF)/epoxy composites is still in early development compared with conventional resin infusion methods. Monticeli et al. [229] predict the flexural strength, modulus, and strain of high-performance 3D-printable CF/epoxy composites using an artificial neural network, analysis of variance, and response surface methodology. The predictions show high reliability with low error, closely matching experimental results. By including different input data, the system can predict various output parameters. Factors such as vacuum pressure, printing speed, curing temperature, and thickness were analyzed, demonstrating efficient fabrication of composite materials with tailored properties.

Malley et al. [230] integrate data analytics with AM to predict the mechanical behavior of samples produced via vat polymerization with varying magnetic particle compositions. A neural network model was developed using mechanical test data from six compositions [231]. The model accurately predicted the mechanical behavior of tested samples and performed well for untested compositions, surpassing traditional data-driven methods. This approach reduces the need for extensive post-manufacturing testing, accelerating product development and improving quality assurance in AM for industrial applications

Griffiths et al. [232] explore the evolution of direct digital and AM from rapid prototyping to rapid production, highlighting its potential for creating personalized, high-quality products with minimal batch sizes. The accessibility of affordable AM machines and open-source software has empowered users, prompting shifts in energy and material consumption patterns. Using a Design of Experiments (DOE) approach, the study optimizes part performance by examining factors such as scrap weight, part weight, energy consumption, and production time. Key findings indicate that optimizing machine parameters can yield desired outcomes, while identical settings across different designs may produce varying results, underscoring the need for design-specific models. The research aims to identify optimal FDM settings for part weight and production time while balancing these with economic factors like energy consumption and scrap weight. Using polylactic acid (PLA) filament for testing, the study analyzes data on weight, build time, and power consumption, employing MiniTab software to visualize parameter interactions through main effects, Pareto, and contour plots. Ultimately, the research contributes valuable datasets for modeling AM processes, facilitating a more accurate assessment of their economic and environmental impacts during the design stage [233].

Table 4 summarizes various studies that utilize ML to predict properties in 3DP.

### 4.2. Process Control and Monitoring

ML enhances process control and monitoring during the 3DP of polymer composites by providing real-time analysis and feedback. This capability helps in maintaining the quality and consistency of printed parts. AI-augmented additive manufacturing (AI2AM) technology was highlighted by Sani et al. [234] and integrates AI-based monitoring and optimization of 3DP parameters to detect and prevent defects, enhance quality and efficiency, and enable more sustainable manufacturing, with a focus on FDM printers and future developments in closed-loop systems.

Real-time defect detection and closed-loop adjustment are essential for ensuring the quality of carbon fiber-reinforced polymer (CFRP) composites in AM. Lu et al. [235] introduce a deep learning-based system for real-time identification and correction of defects in robot-based CFRP AM. The model accurately detects and classifies defects like misalignment and abrasion, while also quantifying their severity through geometric analysis. By integrating this with process parameter adjustments, the system effectively controls defects, achieving what conventional composite fabrication methods cannot.

Narayanan et al. [236] developed a self-monitoring system using real-time camera images, and deep learning detects delamination in FDM 3D-printed parts, while strain measurements predict warping before it occurs. The developed system successfully classifies delamination levels and pre-diagnoses warping, offering potential for automated error detection in various manufacturing processes.

Jin et al. [237] present an automated method for identifying defective 3D-printed polymer parts using images captured during the FFF process. ML (PCA and SVM) and deep learning (CNN) classify parts as good or defective with 98.2% and 99.5% accuracy, respectively, benefiting both manufacturers and hobbyists.

Error detection during extrusion-based AM remains a challenge, with most inspections occurring post-production. Charalampous et al. [238] introduce a vision-based method that compares real-time point cloud data from printed parts to digital 3D models, enabling real-time error detection [239] and performance evaluation to reduce waste and production costs.

An online quality monitoring system using laser scanning detects defects in material extrusion 3DP by comparing surface point clouds with CAD models, and this was investigated by Lin et al. [240]. It reconstructs 3D models of defects, enabling feedback control, and helps reduce material and time waste by determining if the 3D printer should be shut down [241].

The challenge of quality assurance in AM is addressed in [242] by the authors developing an online reinforcement learning (RL) method to detect and mitigate new defects during printing. The method, Continual G-learning, leverages offline knowledge from the literature and online learning during the AM process to minimize required training samples. Applied to a fused filament fabrication (FFF) platform, the method optimally mitigates defects in real time [197,243], demonstrating its effectiveness in both numerical and real-world case studies.

Carrico et al. [244] introduce a new paradigm for manufacturing and controlling soft ionic polymer–metal composite (IPMC) actuators for soft robotics using 3DP. The process creates 3D monolithic IPMC devices with integrated sensors and actuators, and Bayesian optimization is employed to control the actuators, mitigating complex dynamics. The approach improves actuator performance, demonstrated through a modular reconfigurable soft crawling robot, highlighting its potential for more advanced IPMC devices.

Omairi et al. [245] review AI-based predictive models in AM, emphasizing their role in making AM “smart” by improving printability, reducing design complexity, and enhancing real-time control and defect detection. They discuss current trends, research gaps, and opportunities for further collaboration and development in line with Industry 4.0.

Table 5 summarizes the key studies focused on monitoring and adaptive control in AM, detailing their objectives, applied models, and data utilized.

### 4.3. Defect Detection and Failure Prediction

Detecting and predicting defects in printed parts ensure the reliability and performance of polymer composites. ML techniques offer advanced capabilities for identifying and addressing potential issues before they lead to failures. Moreover, predictive models can estimate the likelihood of failure based on historical data and current process conditions. By analyzing factors such as material properties, processing parameters, and environmental conditions, ML can forecast potential issues and recommend preventive measures.

Chen et al. [246] address the challenge of processing large tomography datasets for defect detection in composite materials. Using a micro-CT scan of fiber-reinforced composites, ML models were trained to detect defects. The binarized statistical image features (BSIF) method was applied to compress images without losing defect information. The convolutional neural network (CNN) model achieved high accuracy with a mean square error of 0.001 in fiber orientation prediction, enabling effective defect detection.

Moreover, Chen [247] highlights the unique microstructural signatures in 3D-printed glass fiber-reinforced polymer (GFRP) composites, which can be analyzed using ML to reverse-engineer the tool path. By processing micro-CT images with the BSIF method for data compression, ML models were trained to accurately identify the tool path. This approach poses a potential intellectual property risk for AM, as tool paths could be reconstructed from product microstructures.

FFF faces challenges like inconsistent part quality and print repeatability due to manufacturing defects. Goh et al. [248] developed an on-site monitoring system using computer vision and object detection models to detect and correct such defects in real-time. A camera on the print head captures video, which is processed to detect under-extrusion and over-extrusion anomalies. Various YOLO architectures were tested, with the YOLOv3-Tiny and YOLOv4-Tiny models achieving over 80% accuracy. Optimized models reached 89.8% classification accuracy and 70 frames per second inference speed. A correction algorithm was also implemented, allowing real-time defect detection and correction during printing, advancing FFF process reliability.

A hybrid method combining an artificial neural network (ANN) and micromechanics is developed in [249,250,251] for predicting failure in IM7/8552 unidirectional composite lamina under triaxial loading. The ANN, trained with data from a finite element method-based representative volume element (RVE) model, achieves over 97.5% accuracy. This approach reveals an elliptical paraboloid 3D failure surface and can refine existing failure criteria.

Wan et al. [252] suggest a data-driven approach incorporating probability, and micromechanical modeling predicts failure in IM7/8552 unidirectional CFRPs under biaxial stress. Using high-fidelity 3D RVE models and ANN training, the method achieves a mean square error of 0.027% and a mean absolute error of 0.78% for regression, and a 98.1% prediction probability for classification. The ANN predictions align well with Tsai-Wu and Hashin failure criteria.

AM of carbon fiber-reinforced polymer (CFRP) composites allows for complex structures but challenges remain in predicting mechanical properties. A data-driven model [253] predicts flexural strength in continuous carbon fiber-reinforced polymers (CCFRPs) fabricated by fused deposition modeling (FDM), considering design factors like fiber layers, fiber rings, and polymer infill patterns. ML validates these predictions against experimental data.

High-fidelity simulations of composite materials are computationally intensive. Sepasdar et al. [254,255] introduce a deep learning framework using two fully convolutional networks to predict post-failure stress distribution and crack patterns in 2D composites based on microstructures. Trained on 4500 synthetic representations, the framework achieves 90% accuracy, aided by a physics-informed loss function.

Table 6 provides a summary of key studies focused on defect classification and failure prediction, detailing the focus, data used, and applied methods for each research effort.

### 4.4. Customization and Personalization

ML enables the customization and personalization of polymer composite parts, making it possible to tailor products to specific user requirements and applications.

Xue et al. [256] propose an optimization framework using a variational autoencoder (VAE) and Bayesian optimization (BayesOpt) to design mechanical metamaterials with specific macroscopic elastic properties. By reducing the design space, this approach efficiently optimizes multi-material 3D-printed samples, validated through experimental testing.

A deep learning approach with high-order Bézier curves and a hybrid neural network–genetic optimization (NN-GO) method is used by Lee et al. [257] to optimize lattice structures for better weight-to-performance ratios. The design shifts material towards weak joint regions, improving modulus and strength, validated through AM and compression testing.

An inverse design method using artificial neural networks and generative adversarial networks (GANs) efficiently designs architectured composite materials. The method by Qian et al. [258] reduces the need for massive labeled training data while maintaining high performance, achieving a reduction in computational resources.

Multi-material inkjet 3DP enables the personalization of medical devices by combining algorithmic design with selective material deposition. He et al. [259] reduce bacterial biofilm formation and allow for user-defined mechanical properties, providing multifunctional customization through generative design and finite element modeling.

A heterogeneous microstructural design methodology is applied in [260] to elasto-electro-active piezoelectric ceramics for sensing and energy harvesting applications. Using a vision transformer-augmented VAE, the study creates a generative neural network to design 3D microstructures with multifunctional properties, optimizing them during the inference phase.

ML is used to discover novel lattice metamaterials that optimize elastic stiffness and wave speed during impact. Garland’s et al. [261] AI-driven approach works with minimal simulation calls, overcoming challenges in designing materials for high-performance applications involving complex multi-physics interactions.

Table 7 provides an overview of studies that highlight different approaches to optimization and design using ML.

### 4.5. VAT Photopolymerization and ML

Recent advancements in VAT photopolymerization (VP) enable the creation of complex, customizable materials using techniques like SLA. Sachdeva et al. [262] discuss the evolution, trends, challenges, and future directions of AI in 3DP [46], emphasizing its significance in Industry 4.0.

VP excels at processing polymer composites with high filler content. However, increasing the filler volume raises the suspension viscosity, which conflicts with VP’s need for low-viscosity resins. Additionally, factors like filler shape, size, and optical properties affect light interaction. To address these challenges, Nasrin et al. [263] introduce an artificial neural network (ANN)-based classification model to predict the printability of highly filled polymer suspensions in VP. The model, trained on a small dataset, considers both monomodal and bimodal particle distributions and helps map suitable material and process parameters, optimizing printing efficiency and reducing resource usage.

Mechanoluminescent (MechL) materials emit light when subjected to mechanical stimuli, making them promising for structural health monitoring. However, their practical application has been hindered by challenges in producing high-intensity MechL composites and fabricating complex 3D shapes. Jo et al. [264] introduce a novel method for creating SrAl_2_O_4_^2+^, Dy^3+^ particle-based MechL composites using VP 3DP, optimized through ML. A multi-objective Bayesian optimization (MBO) approach with Gaussian process regression (GPR) was employed to fine-tune critical process parameters, including MechL particle content, layer thickness, and cure ratio. This optimization aimed to enhance MechL properties while reducing printing time. The GPR model captured the complex input–output relationships, allowing for the identification of Pareto-optimal solutions that improved the performance of MechL specimens. Additionally, a micromechanical analysis method was developed to examine the influence of MechL particle volume fraction on MechL intensity. The optimized VP process was validated through practical tests on MechL-based stress sensors and mechanical components.

Frumosu et al. [265] focus on enhancing automation in AM by developing an online monitoring system for bottom-up photopolymerization AM (VPP) processes. The system uses sensor data to detect detachment errors in real time, which can lead to wasted material and time if unnoticed. The monitoring procedure involves an offline phase for training a predictive model and an online phase using a control chart to track detachment predictions. This approach improves process efficiency and can be adapted to other AM technologies, contributing to the shift from prototyping to continuous production.

Shan et al. [266] introduce a low-cost smart resin vat for real-time monitoring of VP 3DP to improve quality control, reliability, and minimize waste. Thermistors placed along the vat’s edges detect heat changes during polymerization, allowing temperature profiles to reflect the curing patterns. ML algorithms are used to assess printing status, with a Failure Index to detect active or terminated prints. Gaussian process regression predicts the printing area based on temperature data. The system successfully detects printing issues, such as failures and missing features, and can be applied across various VP methods. Limitations and future improvements are discussed.

Cao et al. [267] present a method for predicting the optimal waiting time during bottom-up VP 3DP. The waiting time ensures that the printer’s release membrane recovers and the resin becomes stationary between layers, improving print quality. The proposed method, called WTP-VP, uses multilayer perceptrons (MLPs) to predict waiting time based on resin flow and pressure data. This approach reduces waiting time by 47% and overall printing time by 25%, while maintaining surface quality. The method is efficient for real-time predictions in complex topologies and requires fewer data than conventional models.

DLP VP is widely used in AM for creating diverse products layer by layer. A key performance metric is the degree of curing (DoC), which affects material properties like density and elasticity. Current in situ monitoring methods, such as FT-IR, are limited to single-point measurements and can disrupt the process. Zhang et al. [268] introduce a non-invasive, full-field interferometric curing monitoring (ICM) method for real-time tracking of curing dynamics in DLP-VPP. Using a physics-based sensor model and ML, the ICM system estimates refractive index changes to predict DoC, enabling improved process control and print quality.

Table 8 summarizes recent studies that explore the integration of ML models in predicting printability, detecting defects, and optimizing operational parameters in VP-based AM systems.

## 5. Application of ML in 4DP of Polymer Composites

Four-dimensional printing is an advanced manufacturing technique where 3D-printed objects transform over time in response to external stimuli such as temperature, moisture, light, or magnetic fields. When applied to polymer composites, this technique enables the creation of dynamic structures that can adapt their shape or properties post-fabrication. ML has become instrumental in enhancing the capabilities of 4DP of polymer composites, optimizing both the design and functionality of the materials. Since the number of publications on ML in 4DP is relatively small [269], and even fewer focus on polymer composites, this section will review one article at the intersection of these topics, along with a few related studies.

Wang et al. [270] apply ML to predict the hardness of quaternary polymer blends during 3DP, aiming to reduce development costs and speed up multi-material co-blending technology. Using four polymers (PLA, TPU, PETG, ABS), composite materials of varying hardness were created from random three-material combinations. Hyperparameter optimization of five ML algorithms, using particle swarm and genetic algorithms, produced accurate predictive models. A four-in-one mixing extrusion head was built, validating predictions with real measurements. This approach improves the efficiency of multi-material printing design, reducing time and resource costs, with potential applications in high-cost industries like aerospace and biomedical fields.

Sun et al. [271] integrate active composites and 4DP to enable shape transformation in response to environmental stimuli. The process involves using ML and evolutionary algorithms (EAs) to optimize the design of materials with different expansion properties. A recurrent neural network (RNN) model, trained with finite element simulations, predicts forward shape changes, while the ML-EA approach efficiently solves inverse design problems. Combined with computer vision, this method transforms hand-drawn profiles into 4D-printed active beams that morph into desired shapes. The technique offers an efficient design tool for creating complex 4D-printed structures using grayscale digital light processing (g-DLP) [272].

Hamel et al. [273] explore the design of active composites, materials that respond to environmental stimuli, using a ML approach. By combining the finite element method with an evolutionary algorithm, the paper addresses the challenge of optimizing material distribution within 3D-printed active composites to achieve specific shape changes, a process known as 4DP. The composite structures are divided into voxel units made of either passive or active materials, and the optimization is tested through examples to demonstrate the effectiveness of achieving target shapes.

## 6. Transformation of Polymer Composites to Ceramics and Other Materials

The transformation of polymer composites into ceramics and other advanced materials typically involves converting polymer precursors into ceramic forms through methods such as pyrolysis, calcination, or thermal treatment. The inherent properties of polymer composites, including their lightweight nature and flexibility, can be harnessed and enhanced during this transformation, resulting in materials with superior mechanical strength, thermal stability, and resistance to harsh environments. Such transformations not only expand the functional capabilities of the original materials but also pave the way for innovative applications across various fields, including aerospace, electronics, and biomedical engineering. This section explore recent studies associated with the conversion of polymer composites into ceramics and other high-performance materials, highlighting their potential impacts on future technological advancements.

Su et al. [274] present a novel precursor-derived SiOC ceramic (PDC-SiOC) architecture for effective terahertz (THz) electromagnetic interference (EMI) shielding and absorption. The bulk SiOC ceramic absorbs over 93% of THz waves between 1.2 and 1.6 THz. A lightweight honeycomb structure, inspired by moth wings, was fabricated using vat photopolymerization 3DP followed by pyrolysis. This architecture demonstrated a maximum shielding effectiveness of 64.1 dB and a transmissivity below 1.4% from 0.2 to 1.6 THz, absorbing 97–99.8% of THz waves in the same range. Additionally, it exhibited good mechanical properties, with compressive and flexural strengths of 1.2 and 16.5 MPa, and thermal stability up to 1100 °C in inert conditions, highlighting its potential for high-efficiency THz EMI shielding applications.

Lyu et al. [275] present the development of diatom frustule-derived porous silica (DFPS) ceramics, which serve as templates for creating Ti3C2Tx/DFPS composites with exceptional electromagnetic interference (EMI) shielding properties. The composites, hot-pressed at 800 °C, achieved a maximum shielding effectiveness (SE) of 43.2 dB in the X-band and a compressive strength of 67.5 MPa. The hierarchical porous structure enhances electromagnetic energy dissipation through scattering and reflection, making these composites promising for delicate electronic components in the aerospace sector.

Wang et al. [276] focus on the fabrication of short carbon fiber-reinforced silicon carbide (Csf/SiC) ceramic matrix composites (CMCs) through material extrusion (ME) 3DP followed by precursor infiltration and pyrolysis (PIP). The study investigates how solid loading and fiber content affect the microstructure and mechanical properties. Optimal compositions yielded high-performance Csf/SiC CMCs with a bending strength of 212.74 MPa and fracture toughness of 5.84 MPa m^1/2^. The findings contribute valuable insights into the 3DP of fiber-reinforced CMCs.

Sarvestani et al. [277] explore the use of polymer-derived ceramics (PDCs) with enhanced toughness and versatility, fabricated through stereolithography (SLA) using a silicon oxycarbide precursor. Triply periodic minimal surface (TPMS) designs are 3D printed and pyrolyzed to produce intricate ceramic structures. The resulting PDCs exhibit a compressive strength of 2.2 MPa and stiffness of 330 MPa, while maintaining a low density of 0.5 g/cm^3^. The research highlights the potential of low-cost SLA 3DP for creating customized, bio-inspired ceramic architectures.

Jiang et al. [278] develop ultraviolet (UV)-curable polymer precursors and a two-stage pyrolysis strategy to create polymer-derived ceramics (PDCs) with controllable deformation and complex programmable shapes. Despite a low precursor ceramic yield of 13.5 wt% leading to pyrolysis shrinkage, dense, crack-free SiOC ceramics are achieved. The mechanism of deformation during pyrolysis is analyzed, and the study demonstrates a viable approach for producing programmable PDCs through photopolymerization 4DP.

Zhu et al. [279] optimize the formulation of photosensitive resin for polymer-derived ceramics by incorporating h-BN as a two-dimensional filler. The addition of 1 wt% h-BN enhanced the mechanical properties, achieving a bending strength of 252.4 ± 12.2 MPa and a fracture toughness of 2.7 ± 0.2 MPa·m^1/2^ after pyrolysis. Furthermore, the thermal conductivity of the ceramics increased from 0.44 to 5.34 W·m·K^−1^. The findings indicate that introducing h-BN effectively improves the thermal, electrical, and mechanical properties of precursor ceramics.

Young et al. [280] address challenges in 3DP polymer-derived ceramics by evaluating various post-processing methods to enhance pyrolysis outcomes. The approaches include UV surface flood curing, solvent soaking, and intermediate heating, aimed at increasing cross-linking and reducing defects. The results show that post-processing improved the pyrolysis survival rate to 97% and the ceramic yield to 53%, enabling the production of larger, complex turbine vanes.

Bobrin et al. [281] introduce a novel method for fabricating nanostructured carbon–ceramic multi-materials through polymerization-induced microphase separation 3DP. By combining inorganic precursors and acrylonitrile within a photocurable resin, nanostructured materials are created, which transform into a carbon–ceramic matrix upon pyrolysis. The study reveals that the initial resin composition influences the microstructure and properties of the resulting materials, allowing for a combination of ceramic and carbon characteristics, including low thermal conductivity and high electrical conductivity.

The study [282] presents an efficient technique for preparing SiC ceramics using selective laser printing combined with precursor impregnation and pyrolysis (PIP) and liquid phase sintering (LPS). A particle gradation technique was utilized to enhance the green body density, resulting in SiC ceramics with a flexural strength of 150 MPa and a relative density of 98.2%. The findings demonstrate a viable strategy for fabricating high-performance SiC ceramics via selective laser printing.

Wang et al. [283] review advancements in ceramic 3DP technology, highlighting its potential to revolutionize the ceramic industry by enabling the direct manufacturing of intricate designs without molds. The review discusses the benefits of advanced ceramics, including high strength and corrosion resistance, and analyzes various ceramic 3DP techniques. It also addresses the limitations and challenges of these technologies, aiming to provide strategies for the development and market implementation of new ceramic 3DP methods.

Table 9 summarizes the key studies that explore approaches to enhancing the properties and functionalities of PDCs, including their fabrication methods, material compositions, and resultant mechanical and thermal characteristics.

## 7. Challenges and Limitations

**Figure 12:**
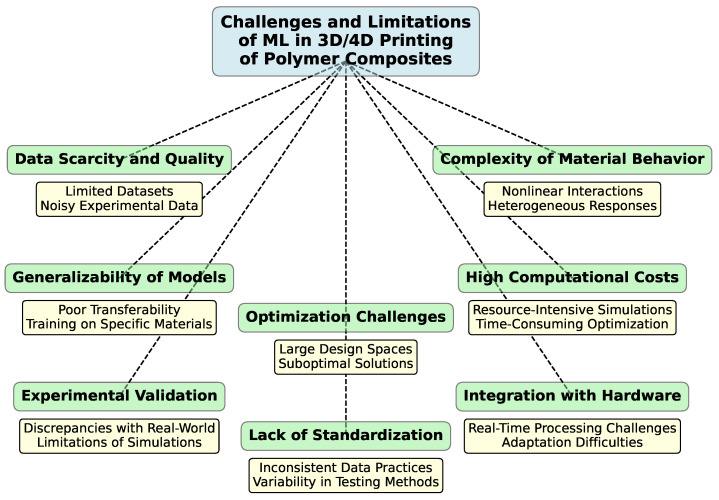
Challenges and limitations associated with applying ML in 3D and 4DP of polymer composites.

The integration of ML into 3D and 4DP of polymer composites holds promise for advancing the field. However, there are several key challenges and limitations that need to be addressed to realize its full potential. Figure 12 illustrates the challenges and limitations associated with applying ML in 3D and 4DP of polymer composites.

One of the foremost challenges is data scarcity and quality [284,285]. ML models typically require large datasets to deliver accurate predictions, but in the field of 3D and 4DP, particularly for polymer composites, such datasets are limited. The data available for material behavior, especially in complex systems involving active composites that respond to environmental stimuli, are often sparse or incomplete. Moreover, experimental data are prone to noise and inconsistencies, making it difficult for ML models to learn effectively and robustly [286].

The complexity of material behavior further complicates the application of ML. Polymer composites, especially when subjected to environmental stimuli in 4DP, exhibit highly nonlinear and heterogeneous behavior [215]. These materials often interact in unpredictable ways when combined, making it difficult for current ML models to accurately predict outcomes [287,288]. For instance, controlling the spatial distribution and phase transitions in multi-material systems, such as mechanical metamaterials or shape-shifting composites, remains a significant challenge due to the complexity of these interactions [289,290,291].

Another limitation is the generalizability of ML models [292,293]. ML models trained on specific material systems or design configurations often fail to generalize well to new materials or different printing methods. This lack of transferability limits the applicability of ML across diverse materials and printing processes [294]. As a result, models that perform well in controlled experimental settings may not be as effective in real-world industrial applications, where new variables are introduced.

High computational costs also present a barrier to the widespread use of ML in 3D and 4DP [295]. Many predictive models rely on computationally expensive simulations, such as the finite element method (FEM), to generate training data and validate results [296]. This is especially problematic for complex inverse design problems, where the optimization of material distributions or structural configurations requires substantial computational resources. The computational burden makes it difficult to apply ML in real-time applications, limiting its scalability for industrial use.

Optimization challenges are another issue, particularly in the context of designing active composites for 4DP. The design process often involves solving inverse problems with large design spaces, such as voxel-based material distributions [297,298]. Even with advanced optimization techniques, like evolutionary algorithms or Bayesian optimization, navigating these vast design spaces can be difficult, and the risk of converging on suboptimal solutions remains high. The complexity of nonlinear material behaviors further exacerbates these challenges, making it harder to find the optimal design.

Furthermore, experimental validation and real-world implementation of ML-driven models are often difficult to achieve. Discrepancies between simulation predictions and real-world outcomes can arise due to variations in material properties, environmental factors, or the limitations of current printing technologies. In practice, ML models that work well in controlled environments may not perform as expected when scaled to industrial applications, where real-time defect detection and quality control are critical [299].

Another practical limitation lies in integrating ML with printing hardware. The real-time monitoring and control of printing processes using ML models require fast and accurate data processing, which is challenging given the high-speed nature of 3DP systems [46,300]. Additionally, adapting existing hardware, such as multi-material extrusion heads, to accommodate ML-driven optimizations can be technically demanding and expensive.

Lastly, the lack of standardization in material characterization, testing methods, and printing protocols across the AM industry further complicates the application of ML [301]. Without standardized datasets and consistent experimental procedures, it becomes difficult to train and compare ML models across different systems or materials, limiting the broader adoption of ML in the field.

For instance, material extrusion (MEX) lacks standardized testing methods tailored to its unique material and process characteristics. Phillips et al. [302] review current practices for preparing tensile test specimens and propose guidelines for future standards. They emphasize the need to account for slicing parameters, specimen geometry, toolpath optimization [303,304], and material specifications to ensure accurate representation of final part properties. Standardizing these factors could improve comparability between studies and support the development of MEX for advanced applications.

Garcia et al. [305] review current design, material, and process standards for AM [306,307], with a focus on mechanical characterization of polymer-based products. They highlight the reliance on standards from other industries, inconsistencies between documents, and the need for clearer guidance. The work highlights the importance of developing AM-specific standards, particularly for mechanical testing, and addresses the disparity between standards for metallic and polymer materials. This review aims to support both researchers and practitioners in navigating the evolving standardization landscape in AM.

## 8. Future Directions

**Figure 13:**
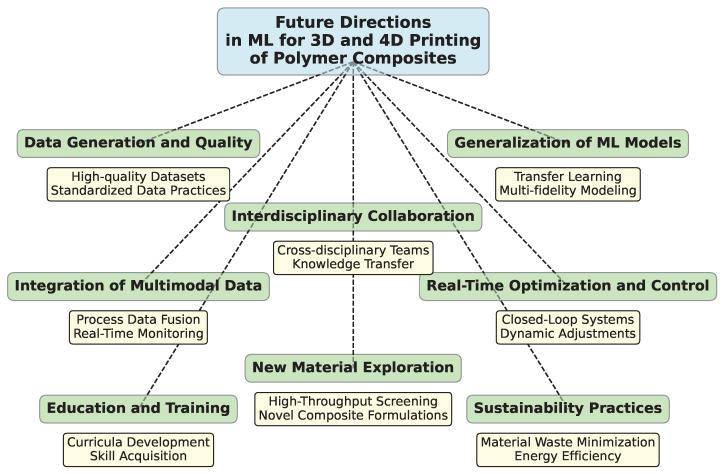
Future directions in ML applications for 3D and 4DP of polymer composites.

As ML continues to evolve, its application in 3D and 4DP of polymer composites presents numerous opportunities for innovation and advancement. To harness the full potential of ML in this field, several key future directions can be explored. Figure 13 illustrates the future directions of ML applications in 3D and 4DP of polymer composites.

One of the most essential areas for future research is the generation of high-quality, diverse datasets [308,309]. This can be achieved through advanced simulation techniques, such as generative adversarial networks (GANs) [310,311], which can synthesize realistic material behavior data. Jabbar et al. [312] review recent advances in using GANs for inverse materials design (IMD), where GANs help discover materials with targeted properties by applying specific constraints. The authors discuss databases, ML criteria, available software tools, and training descriptors for GAN models, highlighting both challenges and future directions in this promising field. On the other hand, Jiang et al. [313] highlight the expanding role of GANs in materials science, covering applications from composition design to microstructure analysis and defect detection. The paper discusses GAN fundamentals, specific use cases, and addresses challenges, underscoring GANs’ potential to drive innovative advancements in materials discovery and optimization.

Additionally, efforts to standardize data collection [314] and sharing practices across research institutions and industries can facilitate greater access to valuable datasets [315]. For instance, Shetty et al. [316] developed a pipeline using NLP and trained a specialized language model, MaterialsBERT, to automatically extract material property data from polymer science abstracts, collecting 300,000 records from 130,000 abstracts in 60 h. The data, accessible at polymerscholar.org, offers insights across applications like fuel cells and solar cells, showcasing the potential of automated literature analysis for materials science.

Future efforts should also focus on developing ML models that exhibit improved generalization capabilities across various materials and printing conditions. This can be achieved by utilizing transfer learning (TL) approaches [317,318], where models trained on one material system can be adapted for others. Tang et al. [319] review TL in AM modeling, emphasizing its potential to improve model quality despite limited data by reusing models across products. They outline TL methods, current applications, and recommendations for effectively applying TL to enhance AM processes.

Multi-fidelity (MF) modeling [320] techniques can also be explored, allowing for the integration of data from different sources (e.g., experimental and simulation data) to enhance model robustness. Nath et al. [321] introduce an MF modeling approach to predict AM outcomes by combining high-fidelity (HF) and low-fidelity (LF) models with experimental data. Using Bayesian calibration, the method improves LF model predictions, demonstrated here for predicting porosity in laser powder bed fusion.

The incorporation of multimodal data is another promising avenue for future research. Integrating process data (temperature, pressure, speed) with material properties and performance outcomes can provide a more comprehensive understanding of the printing process [322]. Petrich et al. [323] propose a supervised machine learning approach for detecting inter-layer flaws in powder bed fusion additive manufacturing (PBFAM) using in situ multimodal sensor data, with 98.5% accuracy in binary flaw classification. Integrating data from multiple sensors (e.g., imagery, acoustic, and multi-spectral) and scan trajectories, the approach successfully correlates in-process sensor data with post-build CT scans, demonstrating enhanced flaw detection performance by fusing sensor modalities.

Advancements in ML algorithms should be geared towards enabling real-time optimization and control of the 3D and 4DP process. This includes developing closed-loop [324] systems, where feedback from in situ sensors informs adaptive control strategies. By continuously learning from ongoing processes, these systems can adjust printing parameters in real time to mitigate defects and enhance material properties, leading to improved efficiency and product quality. Mercado et al. [325] highlight recent efforts in control system improvements and emphasize the potential advantages of closed-loop control for advancing AM precision and reliability.

Exploration of new material systems is another important direction for future research. The application of ML in discovering and optimizing new polymer composite materials for specific applications is promising. By combining ML with high-throughput experimental methods, researchers can rapidly screen and identify novel composite formulations that meet desired performance criteria [326,327]. Nazir et al. [55] provide a summary of recent advancements in multi-material additive manufacturing (MMAM), exploring its applications, design strategies, and challenges across various industries. They identify limitations in existing processes and software, while also discussing future directions and potential strategies to enhance the functionality and mechanical properties of MMAM-fabricated parts.

Interdisciplinary collaboration can facilitate the transfer of knowledge and technology between academia and industry, driving the adoption of ML in real-world manufacturing settings [328]. Park et al. [329] present a methodology for identifying and prioritizing data analytics (DA) opportunities in AM, highlighting the importance of interdisciplinary collaboration. The framework includes a team of experts, a Data Opportunity Knowledge Base (DOKB), and a prioritization tool utilizing Fuzzy-TOPSIS, resulting in the identification and ranking of 264 DA opportunities for the laser powder bed fusion process, ultimately facilitating ongoing collaboration and knowledge sharing within the AM community.

Furthermore, sustainability is an increasingly important consideration in manufacturing. Future research should explore how ML can contribute to sustainable practices in 3D and 4DP [330,331,332], such as optimizing material usage to minimize waste [333], improving energy efficiency [334], and developing biodegradable composites [335,336,337]. Hegab et al. [338] highlight the role of AM in promoting sustainability across various industries, showcasing its benefits in reducing resource depletion, waste, and emissions while improving efficiency in production processes. They discuss the integration of AM within circular economy strategies, identify challenges in its deployment throughout the product life cycle, and emphasize the need for further research on the long-term environmental impacts of AM to encourage its adoption among organizations and policymakers.

As the integration of ML in 3D and 4DP progresses, there will be a growing need for education and training programs that equip researchers and practitioners with the necessary skills and knowledge. Institutions should develop curricula that focus on the intersection of ML, materials science, and AM, ensuring that the next generation of engineers and scientists are prepared to leverage these technologies effectively. Stavropoulos et al. [339] address the lack of expertise hindering the industrial adoption of AM by developing a structured training framework tailored to industry needs. The framework classifies AM into modular educational areas, targeting various professional profiles and emphasizing hands-on practice, while also proposing strategies to enhance accessibility and facilitate the implementation of AM training within the industrial sector.

## 9. Conclusions

The application of ML in 3D and 4DP of polymer composites represents a shift in the landscape of AM. ML applications range from optimizing the printing process to predicting the performance of materials and enhancing design capabilities. By analyzing vast amounts of data generated during the printing process, ML allows for better control of parameters, improved outcomes, and accelerated innovation. Furthermore, as the complexity of designs and materials increases, the role of ML in facilitating rapid prototyping, quality assurance, and customizability will likely grow, positioning it as a cornerstone of future innovations in additive manufacturing. Based on the literature analysis presented in this manuscript, the following is a summarized version in bullet points:

- The integration of ML in real-time monitoring systems (e.g., AI2AM technology for FDM) improves the quality and consistency of printed polymer composites. This shift to smart manufacturing aligns with Industry 4.0 principles, focusing on defect detection and parameter optimization to prevent errors and enhance efficiency.
- Techniques like the deep learning model developed by Lu et al. for detecting defects in carbon fiber-reinforced polymers (CFRPs) showcase the ability of AI to provide real-time geometric analysis and process adjustments. This automation reduces reliance on traditional methods, enhancing overall manufacturing quality.
- Systems like the self-monitoring approach by Narayanan et al. utilize deep learning to detect delamination and predict warping, demonstrating the capability for early error detection. This proactive management improves automated quality control across various manufacturing sectors.
- Jin et al. [340] achieved up to 99.5% accuracy in classifying 3D-printed parts using ML models. This level of precision benefits both large-scale manufacturers and individual users, emphasizing the competitive advantage gained through intelligent quality assurance.
- By employing laser scanning methods (as demonstrated by Lin et al. [240]) to monitor printed surfaces against CAD models, companies can achieve real-time feedback control that reduces waste and prevents unnecessary production runs, leading to more sustainable manufacturing practices.
- The Continual G-learning method for defect detection exemplifies the potential of reinforcement learning to address emerging defects in real time using historical and real-time data, showcasing an advanced adaptive quality control system that requires minimal training samples.
- Innovations like the multi-material inkjet 3DP method described by He et al. for personalizing medical devices highlight the growing trend towards customization, enabling user-defined mechanical properties and multifunctional device design.
- Xue et al.’s variational autoencoder (VAE) framework [256] for designing mechanical metamaterials demonstrates the ability of ML to efficiently customize 3D-printed parts for specific macroscopic elastic properties, fostering innovation in material science and application design.
- The reduction in printing time (up to 25%) and waiting time (47%) achieved through methods like WTP-VP, as described by Cao et al., signify a positive trend towards more environmentally friendly manufacturing processes by minimizing resource usage.
- Chen et al.’s work [21] on tool path identification in GFRP composites highlights the potential risks to intellectual property as reverse-engineering capabilities using ML can reconstruct manufacturing processes from finished products, necessitating enhanced data protection strategies.
- Recent studies highlight advancements in creating polymer-derived ceramics (PDCs) with superior mechanical properties and functionality. For example, Su et al. [274] developed a precursor-derived SiOC ceramic that has the potential for applications in high-efficiency electromagnetic interference (EMI) shielding, particularly in the aerospace and electronics sectors.

## Figures and Tables

**Table 1:** Summary of 3DP techniques with updated terminology according to ASTM 52900:2021.

Technique	Process	Materials	Applications	Advantages	Challenges
Fused Deposition Modeling (FDM) [6,7,8]/Fused Filament Fabrication (FFF) [9,10,11]	Material Extrusion	PLA, ABS, PETG, nylon, composite filaments (e.g., carbon fiber-reinforced)	Prototypes, consumer products, lightweight structures	Low cost, widely accessible, variety of materials available	Rough surface finish, limited mechanical strength
Stereolithography (SLA) [12,13,14]	Vat Photopolymerization	Photopolymer resins (tough, flexible, bio-compatible)	High-detail prototypes, medical/dental models	High precision, smooth surface finish, fine details	Brittle materials, requires post-processing (curing)
Selective Laser Sintering (SLS) [15,16,17]	Powder Bed Fusion	Nylon, polyamide, TPU, composites	Functional prototypes, aerospace/ automotive parts	No support structures needed, strong mechanical properties	Rough surface, more expensive than FDM/SLA
Digital Light Processing (DLP) [18,19,20]	Vat Photopolymerization	Photopolymer resins	Jewelry, dental devices, high-resolution prototypes	Faster than SLA, high resolution	Relies on photosensitive resins, requires post-curing and washing to ensure full polymerization
Multi Jet Fusion (MJF) [21,22,23]	Powder Bed Fusion	Nylon, TPU	Functional parts, small batch manufacturing	Excellent mechanical properties, no supports required	Rough surface finish, requires post-processing (dyeing, etc.)
Material Jetting (PolyJet by Stratasys) [24,25,26]	Material Jetting	Photopolymers (rigid, rubber-like, transparent)	Multi-material prints, medical models, tactile products	High resolution, smooth finish, multi-material printing	Material durability limitations, complex post-processing
Direct Ink Writing (DIW) [27,28,29]	Material Extrusion	Hydrogels, silicones, composite pastes	Soft robotics, biomedical devices, tissue engineering	Can print functional/ biologically active materials	Limited material types, weaker mechanical properties

**Table 2:** Summary of 4DP techniques.

Technique	Process	Materials	Applications	Advantages	Challenges
Shape Memory Polymer (SMP)-Based 4DP [31,32,33,34]	Printed using techniques like FDM, SLA, or SLS, incorporating shape memory polymers that “remember” a programmed shape and return to it when exposed to stimuli (e.g., heat).	Shape memory polymers, composites	Self-assembling structures, biomedical devices (e.g., stents), robotics, adaptive products	Programmable and responsive to stimuli	Limited availability of high-performance SMP materials, complex control over transformations
Hydrogel-Based 4DP [35,36,37,38,39]	Hydrogels are printed using DIW, PolyJet, or SLA, designed to change shape or properties in response to water or humidity.	Hydrogels, stimuli-responsive polymers (e.g., pH-responsive, temperature-responsive)	Tissue scaffolds, drug delivery systems, wearable electronics	Biocompatibility, highly responsive to environmental conditions	Control over swelling, ensuring long-term stability
Stimuli-Responsive Composite-Based 4DP [40,41,42,43,44,45]	Printed using standard 3DP methods (FDM, SLS, etc.) but with composite materials that react to stimuli such as light, magnetic fields, or heat.	Composites with nanoparticles, liquid crystal elastomers, magnetically active particles	Soft robotics, aerospace components, deployable structures	Tailored responses to specific external stimuli	Complex fabrication processes, challenges in controlling transformations

**Table 3:** Summary of recent studies in AM methods.

Reference	Method	Focus	Advantages	Disadvantages
Franco et al. (2024) [63]	FDM	4DP with responsive structures for smart textiles	Enables complex structures; potential for smart applications	Still evolving; challenges in material properties
Subramani et al. (2024) [64]	FDM	Effect of FDM parameters on mechanical properties of ABS components	Identifies optimal settings for improved mechanical properties	Limited to specific materials and printers
Melentiev et al. (2024) [68]	FDM	Improving adhesion in multi-material components using MPAM	Enhanced structural integrity of metalized plastics	Complex multiprocess setup
Bahrami et al. (2024) [72]	FDM	Enhancing wear resistance in ABS through Fe composite filaments	Improved wear performance with optimized parameters	Limitations in wear resistance of pure FDM parts
Hajjaj (2024) [75]	FDM	Comparison of mechanical properties in zirconia restorations	Insights into material performance for dental applications	FDM-printed parts show inferior mechanical properties
Khan et al. (2024) [76]	FFF	Mechanical properties of lightweight polymer structures	Cost-effective and adaptable for different materials	Process parameters can limit mechanical performance
Kariuki et al. (2024) [77]	FFF	Flexural behavior of carbon fiber-reinforced PA12 parts	Optimized parameters enhance mechanical properties	Requires careful selection of printing parameters
Garcia et al. (2024) [79]	FFF	Comparison of FFF with MIM and PM on stainless steel properties	Superior tribocorrosion resistance in FFF parts	Variability in mechanical properties across methods
Kalinke et al. (2024) [80]	FFF	Sustainable practices in 3DP	Focus on recycling and environmental impact	Challenges in material selection for sustainability
Sun et al. (2024) [97]	SLA	Hydrogel-based electronics for wearable devices	High conductivity and flexibility in applications	Low stretchability in traditional hydrogels
Zhou et al. (2024) [99]	SLA	Producing advanced ceramic objects with complex geometries	High resolution and quality for intricate designs	Thermal debinding can lead to defects
Kulkarni et al. (2024) [101]	SLA	Printing polymer nanocomposites with stimuli-responsive materials	Enhanced mechanical properties with effective particle dispersion	Limited by material formulation options
Curti et al. (2024) [103]	SLA	Personalized medicine through SLA	High resolution suitable for drug formulation	Limited specialized excipients for pharmaceutical SLA
Song et al. (2024) [126]	SLS	Medical engineering applications for implants and prosthetics	Precise production of complex biomedical products	High setup costs and limited material options
Azam et al. (2024) [127]	SLS	Electrically conductive polymer composites	High performance for advanced applications	Process complexity can affect production speed
Han et al. (2024) [128]	SLS	Enhancing properties of PA12 composites with CNTs	Improved mechanical and functional properties	Requires careful control of material interactions
Zhang et al. (2024) [120]	SLS	Impact of process parameters on CF/PEEK composites	Excellent mechanical properties for advanced applications	Complex relationships between parameters can complicate optimization
Melentiev et al. (2024) [139]	DLP	LMAM	Produces intricate structures with high resolution and no support structures; ideal for small, precise devices	Limited to specific applications, high dependence on materials
Guo et al. (2024) [137]	DLP	MWCNT-reinforced photosensitive resin	Enhances mechanical and electrical properties; optimized distribution through treatment	Limited research on integration into PR systems
Senthooran et al. (2024) [133]	DLP	Enhancement of mechanical and thermal properties using mica	Improvements in tensile and flexural strength	Material handling and dispersion challenges
Wang et al. (2023) [140]	DLP	Flexible multistage honeycomb structure absorbers	Exceptional EM wave absorption properties; lightweight and flexible	Limited application scope and complexity of design
Alomarah et al. (2024) [143]	MJF	Hybrid auxetic structures in AM	Robust specimens with high dimensional accuracy	Lower print quality with certain techniques like FFF
Tan et al. (2024) [117]	MJF	Simulating fiber-reinforced polymer composites	Improved understanding of pore formation; better material performance predictions	Complexity in modeling and simulation accuracy
Kafi et al. (2024) [142]	MJF	Absorption phenomena in printed polypropylene (PP)	Insights into porosity and mechanical properties	Variability in build orientation effects on performance
Conway et al. (2024) [161]	MJF	Geometric accuracy in surgical guides	High repeatability and accuracy in personalized surgical tools	Time-consuming measurement processes for validation
Patpatiya (2024) [162]	PolyJet	Advanced multi-material structures	Exceptional precision in complex geometries; versatile material options	Challenges with material performance and interfacial bonding
Azpiazu et al. (2024) [163]	PolyJet	Flexural strength in dental prostheses	Significant effects of surface finishing on strength	Thermocycling negatively impacts strength across protocols
Krause et al. (2024) [164]	PolyJet	Microfluidic channels in 3DP	High reproducibility and accuracy for fine features	Limited effective feature sizes for optimal results
Aberdeen et al. (2024) [165]	PolyJet	Bi-material coupons and mechanical failure dynamics	Insights into interface design for multi-material applications	Challenges with interface strength despite geometric improvements
Abas et al. (2024) [179]	DIW	Layer-by-layer deposition of functional materials	Excellent adaptability to flexible substrates and multi-material printing	Limited ink variety restricts commercial applications
Bhardwaj et al. (2024) [173]	DIW	Hydrogel inks in biostructures	Advancements in 4DP for healthcare applications	Challenges in ink consistency and availability
Baniasadi et al. (2024) [181]	DIW	Applications in tissue engineering and robotics	Flexible manufacturing for complex geometries	Limited material choices can restrict applications
Van et al. (2024) [182]	DIW	Conductive fillers in printed electronics	Enables high-resolution printing for sensors and devices	Challenges with material consistency and process optimization
Khalid et al. (2022) [186]	4DP	Shape memory polymers (SMPs)	Responsive structures for various engineering applications	Mechanical property limitations and design flexibility issues
Qiu et al. (2024) [187]	4DP	Fiber-reinforced polymer composites (FRPCs)	Enhanced mechanical performance and actuation capabilities	Challenges in material composition and manufacturing processes
Yan et al. (2023) [33]	4DP	SMP composites	Advances in biomedical applications; unique structural designs	Challenges in achieving consistent properties across applications

**Table 4:** Summary of research on ML applications for predicting properties in 3DP.

Reference	Focus	Data Info	Applied Method
Elbadawi et al. [221]	AI/ML for enhancing FDM 3DP and filament production	N/A	Developed M3DISEEN web-based software
Ong et al. [225]	Balancing dataset for HME and FDM formulations	1594 formulations from in-house and literature data	ML models for predicting printability, mechanical characteristics
Peloquin et al. [226]	Mechanical properties of 3D-printed gyroid lattices	Experimental data for gyroid lattices	Kernel ridge regression ML model
Khusheef et al. [228]	Predicting mechanical properties in FDM	In-process sensing data including IMU and thermal camera	Hybrid deep learning models (CNN-LSTM)
Monticeli et al. [229]	Predicting properties of CF/epoxy composites	Various input parameters: vacuum pressure, printing speed, etc.	Artificial neural network, ANOVA, response surface methodology
Malley et al. [230]	Predicting mechanical behavior in vat polymerization	Mechanical test data from six compositions	Neural network model
Griffiths et al. [232]	Optimizing part production in AM considering environmental impact	Analyzed scrap weight, energy use, production time	Design of Experiments approach

**Table 5:** Summary of studies on real-time monitoring and adaptive control in AM.

Reference	Focus	Applied Model	Data Info
Lu et al. (2023) [235]	Real-time defect identification in CFRP AM	Deep learning system for defect detection	Utilizes geometric analysis of defect severity based on camera feed images from the printing process.
Narayanan et al. (2019) [236]	Self-monitoring system for FDM	Deep learning for delamination detection	Employs real-time camera images and strain measurements from printed parts to predict warping.
Jin et al. (2020) [237]	Automated defect identification in 3DP	ML (PCA, SVM) and deep learning (CNN)	Utilizes image data captured during the FFF process for classification of parts as good or defective.
Charalampous et al. (2021) [238,239]	Vision-based error detection during extrusion	Comparison of real-time point clouds with digital models	Compares 3D-scanned point cloud data from printed parts against digital models to identify discrepancies.
Lin et al. (2019) [240,241]	Online defect detection via laser scanning	Surface point cloud comparison with CAD models	Involves laser scanning data for 3D reconstruction of defects compared with CAD models for feedback control.
Chung et al. (2022) [197,242,243]	Quality assurance via reinforcement learning	Continual G-learning method	Uses historical data and online learning during AM processes to minimize defects based on previously learned patterns.
Carrico et al. (2019) [244]	Control of soft ionic polymer–metal composite actuators	Bayesian optimization for actuator control	Collects performance data from integrated sensors and actuators to optimize control parameters in real time.
Omairi et al. (2021) [245]	AI-based predictive models in AM	Review of predictive models	Analyzes data from various studies to identify trends and gaps in AI applications for improving AM processes.

**Table 6:** Overview of studies focusing on defect classification and failure prediction in polymer composites AM using ML techniques.

Reference	Focus	Data Info	Applied Method
Chen et al. [246]	Defect detection in composite materials using tomography data	Micro-CT scans of fiber-reinforced composites	ML models with binarized statistical image features (BSIF) and CNN
Chen [247]	Tool path analysis in 3D-printed GFRP composites	Micro-CT images for GFRP composites	ML models trained on BSIF-compressed data
Goh et al. [248]	Real-time defect detection in FFF	Video captured from print head	On-site monitoring system using computer vision and YOLO architectures
Chen et al. [249]	Predicting failure in composite lamina under triaxial loading	Data from finite element method-based RVE model	Hybrid method combining ANN and micromechanics
Wan et al. [252]	Predicting failure in CFRPs under biaxial stress	High-fidelity 3D RVE models	Data-driven approach with ANN and micromechanical modeling
Fontes et al. [253]	Predicting flexural strength in CCFRPs fabricated by FDM	Design factors like fiber layers and polymer infill patterns	Data-driven ML model
Sepasdar et al. [254]	Predicting stress distribution and crack patterns in composites	4500 synthetic representations of microstructures	Deep learning framework with fully convolutional networks

**Table 7:** Overview of studies focusing on optimization and design methodologies in AM using ML techniques.

Reference	Focus	Data Info	Applied Method
Xue et al. [256]	Designing mechanical metamaterials with specific elastic properties	Multi-material 3D-printed samples	Optimization framework using variational autoencoder (VAE) and Bayesian optimization (BayesOpt)
Lee et al. [257]	Optimizing lattice structures for weight-to-performance ratios	Lattice structures designed through AM	Deep learning with high-order Bézier curves and hybrid neural network-genetic optimization (NN-GO)
Qian et al. [258]	Inverse design of architectured composite materials	Labeled training data for neural networks	Artificial neural networks and generative adversarial networks (GANs)
He et al. [259]	Personalizing medical devices with multi-material printing	Algorithmic design combined with selective material deposition	Generative design and finite element modeling to reduce bacterial biofilm formation
Hashemi et al. [260]	Designing elasto-electro-active piezoelectric ceramics	Microstructural design methodology for multifunctional properties	Vision transformer-augmented VAE for generative neural network design
Garland et al. [261]	Discovering novel lattice metamaterials	Optimization for elastic stiffness and wave speed in impact scenarios	AI-driven approach with minimal simulation calls

**Table 8:** Overview of studies focusing on defect classification, failure prediction, and process optimization in VP-based AM.

Reference	Focus	Data Info	Applied Method
Nasrin et al. [263]	Predicting printability of highly filled polymer suspensions in VP	Small dataset on polymer suspensions with monomodal and bimodal particle distributions	ANN-based classification model for mapping material and process parameters
Jo et al. [264]	Optimizing MechL composites using VP 3DP for structural health monitoring	Data on MechL particle content, layer thickness, and cure ratio	Multi-objective Bayesian optimization with GPR; micromechanical analysis
Frumosu et al. [265]	Online monitoring system for bottom-up photopolymerization AM (VPP) to detect detachment errors	Sensor data from bottom-up VPP processes	Predictive model using a control chart for real-time error detection
Shan et al. [266]	Real-time monitoring system for VP 3DP to improve quality control	Temperature data from thermistors placed along the vat edges	ML algorithms with Gaussian process regression and Failure Index to detect print issues
Cao et al. [267]	Predicting optimal waiting time in bottom-up VP 3DP to improve print quality	Resin flow and pressure data	Multilayer perceptrons (MLPs) for predicting waiting time, and reducing printing and waiting times
Zhang et al. [268]	Real-time tracking of curing dynamics in DLP-VPP using a non-invasive method	Full-field interferometric data on refractive index changes	Physics-based sensor model with ML to estimate degree of curing (DoC)

**Table 9:** Summary of recent studies on polymer-derived ceramics.

Reference	Focus	Materials	Methods	Results
Su et al. [274]	THz EMI shielding and absorption	Precursor-derived SiOC ceramic (PDC-SiOC)	Vat photopolymerization 3DP followed by pyrolysis	Absorbs >93% of THz waves (1.2–1.6 THz), SE of 64.1 dB, compressive strength of 1.2 MPa, thermal stability to 1100 °C.
Lyu et al. [275]	EMI shielding properties of composites	Diatom frustule-derived porous silica (DFPS), Ti3C2Tx	Hot-pressing at 800 °C	SE of 43.2 dB in X-band, compressive strength of 67.5 MPa, promising for aerospace applications.
Wang et al. [276]	Short carbon fiber-reinforced SiC CMCs	Short carbon fiber, SiC	Material extrusion 3DP, precursor infiltration and pyrolysis (PIP)	Bending strength of 212.74 MPa, fracture toughness of 5.84 MPa m^1/2^.
Sarvestani et al. [277]	Enhanced toughness and versatility in ceramics	Polymer-derived ceramics (PDCs)	Stereolithography (SLA) using SiOC precursor	Compressive strength of 2.2 MPa, stiffness of 330 MPa, density of 0.5 g/cm^3^.
Jiang et al. [278]	Programmable shapes in polymer-derived ceramics	UV-curable polymer precursors	Two-stage pyrolysis strategy	Achieved crack-free SiOC ceramics, despite 59.91% shrinkage; demonstrated programmable shape capability.
Zhu et al. [279]	Optimization of resin formulation for ceramics	Photosensitive resin, h-BN	Incorporation of h-BN in resin formulation	Bending strength of 252.4 ± 12.2 MPa, fracture toughness of 2.7 ± 0.2 MPa·m^1/2^, thermal conductivity improved to 5.34 W·m^−1^·K^−1^.
Young et al. [280]	Post-processing methods for improved pyrolysis	Polymer-derived ceramics	Various post-processing techniques	Pyrolysis survival rate of 97%, ceramic yield of 53%, enabling larger turbine vanes production.
Bobrin et al. [281]	Fabrication of nanostructured carbon–ceramic multi-materials	Inorganic precursors, acrylonitrile	Polymerization-induced microphase separation 3DP	Revealed influence of resin composition on microstructure; combined ceramic and carbon properties achieved.
Wang et al. [282]	Efficient SiC ceramics preparation	SiC ceramics	Selective laser printing, precursor impregnation and pyrolysis (PIP), liquid phase sintering (LPS)	Flexural strength of 150 MPa, relative density of 98.2%.

## Data Availability

Data are contained within the article.
